# Causes of death among older children and adolescents (5–19 years) in the Magu Health and Demographic Surveillance Study, Tanzania, 1995–2022

**DOI:** 10.1080/16549716.2024.2425470

**Published:** 2024-11-07

**Authors:** Sophia Kagoye, Milly Marston, Yasson Abha, Eveline T. Konje, Mark Urassa, Jim Todd, Ties Boerma

**Affiliations:** a Department of Sexual and Reproductive Health, National Institute for Medical Research, Mwanza Research Centre, Mwanza, Tanzania; b Department of Epidemiology and Biostatistics, Catholic University of Health and Allied Sciences, Mwanza, Tanzania; c Department of Population Health, London School of Hygiene and Tropical Medicine, London, UK; d Community Health Science, University of Manitoba, Winnipeg, MB, Canada

**Keywords:** Cause-specific mortality, 5–19, place of death, verbal autopsy, sub-Saharan Africa

## Abstract

**Background:**

Population data on mortality and causes of death among 5–19-year-olds are limited.

**Objectives:**

To assess levels, trends, and risk factors of cause-specific mortality and place at death among 5–19-year-olds in Tanzania (1995–2022).

**Methods:**

Using longitudinal data from the Magu Health and Demographic Surveillance System in northwest Tanzania, we identified leading causes of death among 5–19-year-olds from verbal autopsy interviews, using physician review and a Bayesian probabilistic model (InSilicoVA). We analyzed trends in cause and place of death using three periods: 1995–2004, 2005–2014 and 2015–2022, and assessed risk factors in a Cox-proportional hazards model. We compared the results with children aged 1–4 years and global estimates for Tanzania.

**Results:**

Between 1995 and 2022, communicable disease mortality decreased by 73%, similar to the 76% decline among 1–4-year-olds. This decline in communicable disease mortality drove all-cause mortality declines of 43% and 48% among 5–14- and 15–19-year-olds, respectively. Non-communicable diseases and injuries gained importance, with their relative share of all deaths increasing from 15% in 1995–2004 to 58% in 2015–2022. Mortality risks were significantly higher among boys (particularly for injuries), those residing in rural areas (for non-communicable diseases), and those from the poorest households (for communicable diseases). By 2015–2022, 48% of 5–14 and 42% of 15–19-year-olds died in health facilities, up from 25% in 1995–2002.

**Conclusions:**

Since 1995, the decline in communicable disease mortality drove a major all-cause mortality reduction among 5–19-year-olds. Further progress will depend on continued reduction in communicable disease mortality, particularly among the poorest, and effectively addressing non-communicable and injury mortality.

## Paper context

- **Main findings:** Between 1995 and 2022, all-cause mortality among 5–19-year-olds declined by over 40%, primarily driven by a reduction in communicable diseases similar to that seen at 1–4 years; this has resulted in a greater prominence of mortality due to non-communicable diseases and injuries.
- **Added knowledge:** The 28 years of mortality data offer valuable insights into long-term changes in causes of death for the often-overlooked age groups of 5–14 and 15–19 in Tanzania and sub-Saharan Africa, showing the changes in cause of death structure over time, existence of cause-specific inequalities by wealth and the importance of empirical data.
- **Global health impact for policy and action:** The observed levels, trends and inequalities in causes of death indicate that further progress in reducing mortality among older children and adolescents depends on a comprehensive health strategy that addresses all major causes with a focus on communicable diseases among the poorest populations.

## Background

An estimated 1,300,000 older children and adolescents aged 5–19 years worldwide died in 2022, with more than half of these deaths occurring in sub-Saharan Africa [1]. The causes of death in this region are not well documented due to inadequate civil registration and vital statistics systems [2,3].

In Tanzania, policies and programs have mainly focused on reducing under-5 mortality, with over 70% reduction since 1990 observed in the 2022 Tanzania Demographic and Health Survey (TDHS) [4]. Mortality and health among children 5–19 years have become more prominent in recent years, as shown in the most recent National Strategic Plan for Reproductive, Maternal, Newborn, Child, Adolescent Health and Nutrition (2021/2022–2025/2026) (One Plan III), aiming to monitor causes of death among children beyond 5 years [5]. Data on mortality levels and causes in this age group are limited, yet having baseline information on the burden of disease is essential for policymakers to ensure appropriate resource allocations, prioritization of interventions and planning for their delivery [5].

In low- and lower-middle-income-countries such as Tanzania, where death registration systems are incomplete, monitoring of mortality and causes of death at a population level is challenging [2]. Empirical data on cause of death can be obtained from health facility studies [6,7]. However, such data are likely to be biased due to the fact that a considerable proportion of deaths occurs outside of health care settings [8]. Population data can also be collected through an interview with relatives of the deceased, known as verbal autopsy (VA) [9–11]. Several methods are available to ascertain the probable cause of death from VA data, including physician review [12] and computer algorithms such as Tariff, InterVA, and InsilicoVA [13–15].

Magu Health and Demographic Surveillance Study (HDSS) in rural northwest Tanzania has been in operation since 1994, collecting data longitudinally on vital events and cause of death through VA [16,17]. In a previous paper, we showed that mortality among older children and adolescents declined by more than 40% during 1995–2022 [18]. In this study, we analyze the levels, trends and risk factors in the cause of death and the transition in the place of death among 5–19-year-olds during the same period. We also compare the disease burden at 5–19 years with younger children aged 1–4 years and with the global estimates for the same age group from Tanzania.

## Methods

### Study setting

Data for this analysis were obtained from a population-based longitudinal design implemented in the Magu HDSS from 1995 to 2022. Magu HDSS was established in 1994 and is located in Magu district, Mwanza region, northwestern Tanzania. The HDSS was initially established with the primary goal to describe the course and determinants of HIV infection. The demographic surveillance system served to monitor mortality by age, sex and cause and was complemented by surveys on HIV epidemiology at multi-year intervals and in-depth studies [16,17].

The area initially comprised seven contiguous villages and was administratively expanded to nine villages without changing HDSS external boundaries. The HDSS lies approximately 20 km from the regional capital city of Mwanza and includes a semi-urban trading center (two adjacent villages, divided into four in 2018) and rural villages. A baseline census was carried out in 1994. Data on vital events (births, deaths, pregnancy and in/out-migration) were collected, on average, every 8 months until 2022, making a total of 40 rounds. Information on the cause of death through VA interviews was collected for all deaths identified in a follow-up visit. The population size in 2022 was about 54,000. Further information on the research activities at Magu HDSS is reported elsewhere [16,17].

### Data collection

Data were collected by trained local staff. A standard structured interviewer-administered questionnaire was used to collect data on vital events (birth, death, pregnancy status, and migration). A trained clinical officer conducted a follow-up visit for the VA interviews. VA is based on a standardized interview conducted with relatives or others who have detailed knowledge of the overall circumstances, signs, and symptoms leading to death.

For children and adolescents, information for the VA was collected from the parents or close relatives. VA interviews were conducted at least 1 month and on average 3 months after death. The absence of a respondent at recurrent home visits (at least two) was recorded as non-response.

The VA tools include sections on the age of the deceased, signs and symptoms of the illness, any available health records, preventive factors and the narration of the illness that led to death, and health care seeking for the illness and related constraints. Different versions of the VA questionnaire have been used: a locally developed questionnaire between 1995 and 2002 [16], followed by an INDEPTH (The International Network for the Demographic Evaluation of Populations and their Health) standard questionnaire between 2003 and 2007 [19], and the standard WHO questionnaire from 2008 [20–22]. The narrations of the illness that led to death were reported from 2008 onwards [20–22] Paper-based questionnaires were used until 2012; thereafter, electronic questionnaires were used. All questionnaires were in Swahili, but interviewers used the local vernacular (Sukuma) when needed.

VA was implemented regularly in all time periods except for most of 2003 and 2004. After data quality control, the questionnaires were standardized into a data specification provided by the ALPHA Network (ALPHA: Analysing Longitudinal Population-based HIV/AIDS data on Africa) (Appendix 2). For this study, we used the questionnaires for children (for ages 1–12 years) and adults (for ages 13 and above) as recommended by WHO [20–22].

### Cause of death assignment

The cause of death assignment from the standardized verbal autopsy data was assigned using two methods: physician review and InsilicoVA computer algorithm [15].

The standardized data were converted into inputs required by a computer algorithm known as InsilicoVA to assign a cause of death. The InsilicoVA algorithm uses the Open VA package in R to ascertain the probable cause of death based on ICD-10 principles. InsilicoVA is a Bayesian probabilistic model that identifies the joint distribution of individual cause of death and population-level cause-specific mortality fractions most consistent with the VA signs and symptoms recorded for a group of deaths. Using the cause-specific fractions for each death, InsilicoVA produces a probability distribution for each cause of death for each individual, the largest of which is taken as the most likely cause of death. The individual cause-specific mortality fractions are aggregated over all deaths to give a cause-specific mortality fraction for the population [15], which describes the percentage of deaths attributable to each cause in a defined group of deaths.

Two local physicians (SK and YA) independently reviewed available data on symptoms and narrations for the physician review and provided a single underlying cause of death. Based on the diagnoses by InSilicoVA and the physician reviews, the final cause of death was determined by a rule of majority (2 or 3); if all three diagnoses were different, the cause of death was coded as indeterminate. The agreement between InsilicoVA and the physician’s cause of death was 52%, while the agreement between the two physicians was 80%. We present the distribution of causes excluding indeterminate causes, which implied that we assumed that the cause distribution for deaths with indeterminate causes was similar to those with known causes. Specific causes of death were classified into broad cause of death groups: communicable, non-communicable, and injuries. The specific classification of the causes of death according to the ICD 10 classification is detailed in Appendix 1 Table S1.

### Statistical analysis

Data were analyzed using Stata version 18.0 [23]. Using the annual residency data from January 1995 to December 2022, we calculated mortality rates as the number of deaths in the HDSS in a given period divided by the number of person-years lived in the HDSS in the same period for each defined age group (5–14 and 15–19 years). The period between out-migration and in-migration was excluded from the denominator if an in-migration followed an out-migration. In order to have adequate numbers of deaths, we presented our findings in three nine-year periods (1995–2004, 2005–2014 and 2015–2022).

We calculated cause-specific mortality fractions disaggregated by sex for 5–14- and 15–19-year age groups. These fractions were applied to the all-cause mortality rates in the HDSS area to obtain cause-specific mortality per 10,000 population, assuming that the distribution of cause of death for those with VA is the same as among those without VA in the same age group and period. We also compared the cause of death distributions among 5–19-year-olds with those among younger children aged 1–4 years which experienced an all-cause mortality decline about two times greater than older children [18]. Also, we compared 5–14 cause of death distribution in the population data from Magu HDSS, which has been considered typical for Tanzania as a whole [17], with the global estimates for Tanzania from United Nations Inter-Agency Group for Child Mortality Estimation (UN IGME) [1] and Global Burden of Disease (GBD) [24].

Further, analyses were disaggregated by sex, place of residence (semi-urban/rural), and household wealth, as the main variables available through the surveillance system. In general, from literature, rural residence and household wealth are associated with poorer survival rates, though most literature focuses on children under the age of five [25–27]. A few studies have analyzed cause-specific inequalities among 5–19-year olds [9,10,25,28] Data on household structure and assets – including household building materials, the primary source of drinking water, use of cooking fuel, in-house assets (e.g. lantern lamp, sofa, bicycle, radio, TV), and livestock (poultry, pigs, donkey cattle, sheep, goats) [29] – were used to calculate a wealth index as a weighted average using principle component analysis [30]. The household wealth scores were grouped into wealth tertiles, with the first tertile representing the 33% poorest and the third representing the 33% richest. We successfully linked the household wealth data with 89% of deaths with verbal autopsy information.

To examine the determinants of cause-specific mortality rates for communicable diseases, non-communicable diseases and injuries among 5–14- and 15–19-year-olds, we used a Cox-proportional hazard model to obtain relative risks (RR) with corresponding 95% confidence intervals (95% CI). We combined all deaths over the whole period and adjusted for the time period, sex, area of residence, and wealth tertiles as guided by previous studies in low- and middle-income countries [9,10,25,28]. The proportional hazards assumption was tested using scaled Schoenfeld residuals, and the overall goodness-of-fit of the model was assessed using Cox-Snell residuals, by plotting the cumulative hazard versus Cox-Snell residuals [31,32].

Data on the place of death were available from the VA interviews. We assessed the proportions occurring in health facilities (hospital/health centre), at home, traditional healer’s place or other location. The latter included on route to/from health facility.

## Results

### Background characteristics of deaths

The background characteristics of deaths among 5–19-year-olds in Magu HDSS during the study period (1995–2022) are presented in Table 1. There were 635 deaths among older children and adolescents aged 5–19 years, with the majority of these deaths (71.8%) occurring among 5–14-year-olds. More than half (57.8%) were males, and about one-third (67.7%) of these deaths occurred in rural areas. Out of 635 deaths among 5–19-year-olds in Magu HDSS, 493 (77.6%) had complete verbal autopsy information with signs and symptoms, and, among those, 57.2% had additional narratives. Further details on VA coverage are presented in appendix 1 on Figure S1 and Table S2.

**Table 1.** Background characteristics of deaths among 5–19-year-olds in Magu HDSS 1995–2022 (N = 635).

	5-14 (*n* = 456)		15-19 (*n* = 138)		Total (*N* = 635)	
	Deaths	Percentage	Deaths	Percentage	Deaths	Percentage
Sex						
Male Female	269 187	59.0 41.0	98 81	54.7 45.3	367 268	57.8 42.2
Calendar year						
1995–2004 2005–2014 2015–2022	157 171 128	34.4 37.5 28.1	69 62 48	38.6 34.6 26.8	226 233 176	35.6 36.7 27.7
Area of residence						
Semi-urban Rural	140 316	30.7 69.3	65 114	36.3 63.9	205 430	32.3 67.7
Wealth tertiles						
Poorest 33% Middle Richest 33% Missing	105 105 105 141	23.0 23.0 23.1 30.9	47 35 41 56	26.3 19.5 22.9 31.3	152 140 146 197	23.9 22.0 23.0 31.0
Complete VA data						
No Yes	101 355	22.1 77.8	41 138	22.9 77.1	142 493	22.4 77.6
Has VA with narrations*						
No Yes	141 214	39.7 60.3	70 68	50.7 49.3	211 282	42.8 57.2

*Denominator: Those with complete VA (5–14 (*n* = 355); 15–19 (*n* = 138); Total (*n* = 493)).

### Cause of death

**Figure 1.**
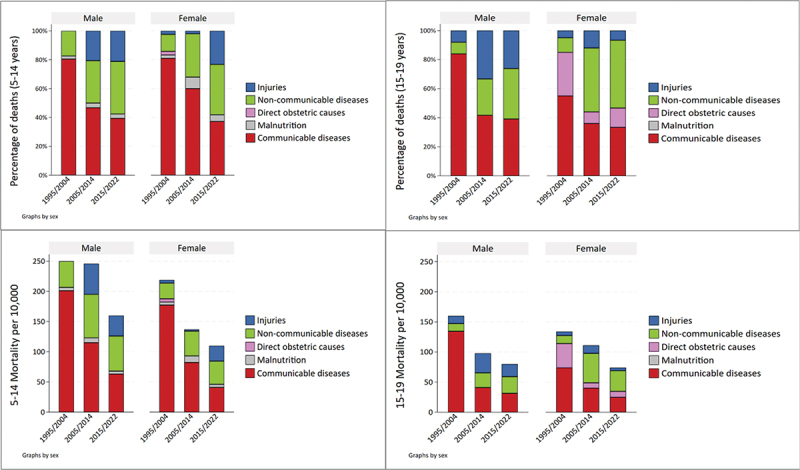
Cause specific mortality fractions (upper panels) and rates (lower panels) by sex among 5–14- and 15–19-year-olds in Magu HDSS from 1995–2022.

The cause of death could be ascertained for 74% of deaths for both 5–14- and 15–19-year-olds (Appendix 1 Table S3). The transition in the distribution of causes of death by the main group is shown in Figure 1. The share of communicable diseases decreased from 80.7% to 38.5% among 5–14-year-olds and from 71.1% to 36.8% among 15–19-year-olds between the first and final periods, respectively. With a decline in communicable disease mortality, the relative share of non-communicable diseases and Injuries increased over time, accounting for 16% during the first period to approximately 58% of deaths among 5–14-year-olds and 15–19-year-olds during the final period.

The share of communicable diseases decreased equally among males and females between the first and final period. Among 15–19-year-olds, non-communicable diseases were more prominent among females than among males (33.4% compared to 22.2%), while injuries were much more common among males (22.2% compared to 8.0%) (Appendix 1 Table S6).

All-cause mortality rates declined by 43% and 48% among 5–14- and 15–19-year-olds between 1995–2004 and 2015–2022. The decline was entirely due to the reduction in mortality due to communicable diseases, which reduced by 73% in both age groups between the first and final periods. There was no decline in non-communicable disease mortality rates for both age groups and minimal change in injury mortality rates between the two last periods, though the rates were considerably higher than in the first period when they were improbably low.

Leading communicable causes of death were malaria and diarrhoeal diseases for 5–14 and HIV/AIDS, tuberculosis and malaria for 15–19-year-olds (Appendix 1 Table S6). Sickle-cell disease was the most common non-communicable cause among children 5–14 years (14% of all deaths), while road traffic accidents and drowning were the most common injuries. The group 1 causes with greater contributions to the mortality decline were HIV/AIDS and tuberculosis, with their combined share decreasing from 10.2% to 3.7% and 26.7% to 5.3% between 1995–2004 and 2015–2022 among 5–14- and 15–19-year-olds, respectively) (Appendix 1 Table S5).

### Cause of death comparison with 1–4 years

**Figure 2.**
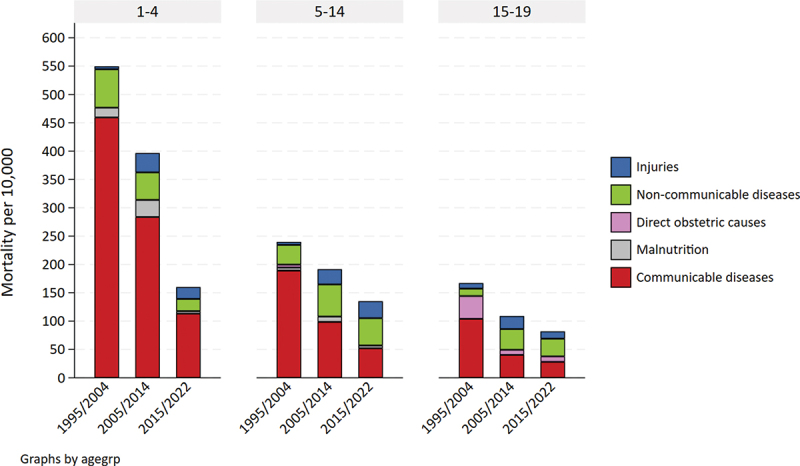
Cause-specific mortality rates per 10,000 person-years among 5–19-year-olds compared to 1–4-year-olds in Magu HDSS (1995–2022).

Among children 1–4 years, the proportion of deaths associated with communicable diseases declined from 84% to 70% between the first and last periods, respectively. The reduction in the all-cause mortality rate in this age group was 70.7%, driven by a decline in communicable disease mortality, which declined by 75.6%. This decline in communicable diseases among 1–4-year-olds was comparable to that among 5–14- and 15–19-year-olds (approximately 73%), but the impact of all-cause rates was greater because of greater prominence of communicable diseases among older children (Figure 2 and Appendix 1 Table S4). The leading communicable cause of death among children aged 1–4 years was acute respiratory tract illnesses, accounting for more than 30% of all causes. The share of non-communicable diseases and injuries was more than two times higher among 5–19-year-olds compared to 1–4-year-olds in the final period (58% compared to 27%) (Appendix 1 Figure S2 and Appendix 1 Table S3).

### Cause of death comparison with global estimates

**Figure 3.**
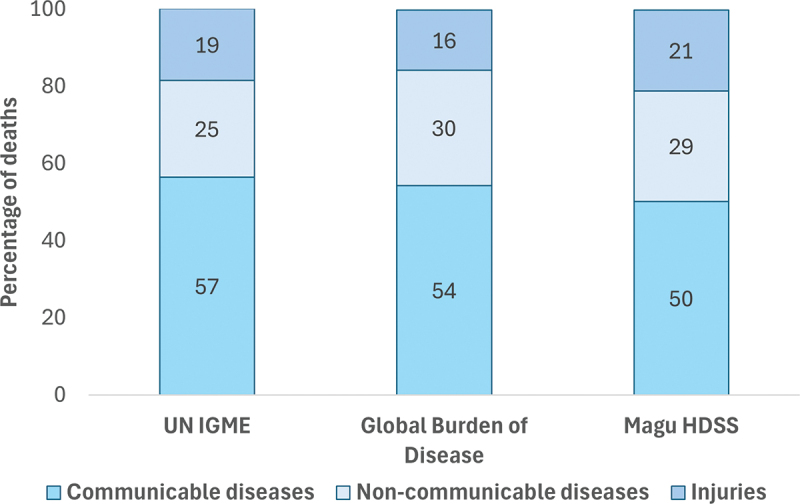
Comparison on cause of death distribution between Magu HDSS and global estimates for Tanzania from UN IGME and Global burden of disease for a period of 2010–2022.

**Figure 4.**
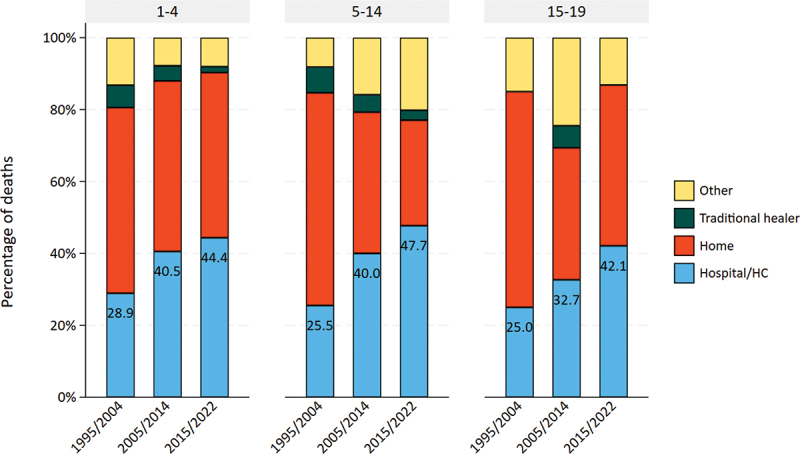
Changes in distribution of place of death among 5–19-year-olds, compared to 1–4 years in magu HDSS (1995–2022).

We compared the cause of death distribution from Magu HDSS with the available global estimates for Tanzania from UN IGME and GBD for the three major groups of cause of death, combined for sex for 5–14-year-olds, for the period 2010–2022 (Figure 4 and Appendix 1 Table S7). Overall, the cause of death distributions in Magu HDSS were comparable to the global estimates for Tanzania (Figure 3). The share of injuries was higher in Magu HDSS compared to the global estimates (Figure 3), mainly driven by road traffic injuries (8.5% in Magu HDSS, compared to 3.6% in UN IGME and 4.3% in GBD). Magu HDSS data also had a much higher share of malaria than the two global estimates (22.6%, compared to 9.9% (UN IGME) and 4.5% (Global burden of disease) but a lower share of HIV/TB, diarrhoea and respiratory diseases (Appendix 1 Table S7)

### Risk factors

After adjusting for sex, area of residence and wealth tertiles, the risk of dying from communicable diseases at 5–14 years decreased by 70% during the final period compared to the first period (AHR: 0.30; 95% CI: 0.19, 0.48) (Table 2). The risk of dying from communicable diseases was 43% higher among males compared to females (AHR: 1.43; 95% CI: 1.03, 1.98). Household wealth was a major risk factor: the mortality risk ratio was 33% and 80% higher among 5–14-year-olds in the middle (AHR: 1.33; 95% CI: 0.82, 2.16) and poorest households (AHR: 1.80; 95% CI: 1.10, 2.95), respectively, compared to those from the richest households.

**Table 2.** Socio-demographic risk factors of cause-specific mortality among 5–14-year-olds in Magu HDSS.

	Communicable diseases	Non-communicable diseases	Injuries
	AHR (95% CI)	AHR (95% CI)	AHR (95% CI)
Calendar time			
1995–2004 2005–2014 2015–2022	1 0.76 (0.53, 1.09) 0.30 (0.19, 0.48)***	1 2.34 (1.26, 4.34)** 1.71 (0.91, 3.24)	1 6.10 (1.42, 26.31)* 5.20 (1.20, 22.59)*
Sex			
Male Female	1.43 (1.03, 1.98)* 1	1.62 (1.07, 2.45)* 1	2.38 (1.18, 4.82)* 1
Area of residence			
Semi-urban Rural	1 0.92 (0.63, 1.37)	1 1.80 (1.06, 3.04)*	1 1.92 (0.83, 4.43)
Wealth tertiles			
Poorest 33% Middle Richest 33%	1.80 (1.10, 2.95)* 1.33 (0.82, 2.16) 1	1.11 (0.61, 2.04) 1.11 (0.62, 2.01) 1	0.90 (0.36, 2.25) 0.80 (0.32, 1.99) 1

*p < 0.05; **p < 0.01; *p* < 0.001; AHR = adjusted hazard ratio.

The risks of dying from non-communicable diseases and injuries were lowest in the first periods but was at similar higher levels in the second and third periods (Table 2). It is likely that the proportions of deaths in these two groups were underestimated for 1995–2002. We observed a significantly higher risk among males compared to females for both non-communicable diseases (AHR: 1.62; 95% CI: 1.07, 2.45) and injuries (AHR: 2.38; 95% CI: 1.18, 4.82). The risk of non-communicable diseases was 80% higher in rural areas compared to urban areas (AHR: 1.80; 95% CI: 1.06, 3.04), but no significant effect was observed by household wealth tertile (Table 2).

Among adolescents aged 15–19-year-olds, after adjusting for sex, area of residence and wealth tertiles, the risk of dying from communicable diseases significantly decreased over time. Compared to 1995–2004, we observed 49% and 67% risk reduction in 2005–2014 (AHR: 0.51; 95% CI: 0.27, 0.98) and 2015–2022 (AHR: 0.33; 95% CI: 0.16, 0.68), respectively. The risk of dying from communicable diseases increased with reducing household wealth, with the poorest having a mortality risk that is more than twice as high as their richest counterparts (AHR: 2.12; 95% CI: 0.95, 4.71) (Table 3).

**Table 3.** Socio-demographic risk factors of cause-specific mortality among 15–19-year-olds in Magu HDSS.

	Communicable diseases	Non-communicable diseases	Injuries
	AHR (95% CI)	AHR (95% CI)	AHR (95% CI)
Calendar time			
1995–2004 2005–2014 2015–2022	1 0.51 (0.27, 0.98)* 0.33 (0.16, 0.68)**	1 2.76 (0.92, 8.25) 1.24 (0.37, 4.12)	1 2.27 (0.62, 8.25) 1.32 (0.33, 5.30)
Sex			
Male Female	1.12 (0.63, 1.99) 1	0.76 (0.36, 1.59) 1	4.58 (1.33, 15.74)* 1
Area of residence			
Semi-urban Rural	1 0.73 (0.37, 1.46)	1 2.92 (1.05, 8.12)*	1 0.72 (0.26, 1.97)
Wealth tertiles			
Poorest 33% Middle Richest 33%	2.12 (0.95, 4.71) 0.71 (0.29, 1.74) 1	0.90 (0.29, 2.78) 1.15 (0.39, 3.37) 1	1.38 (0.32, 5.99) 2.70 (0.80, 9.15) 1

*p < 0.05; **p < 0.01; *p* < 0.001; AHR = adjusted hazard ratio.

The risk of dying from non-communicable diseases was more than twice as high in rural areas compared to urban areas (AHR: 2.92; 95% CI: 1.05, 8.12). The risk of dying from injuries was more than four times higher among males compared to females (AHR: 4.58; 95% CI: 1.33, 15.74) (Table 3).

### Changes in distribution of place of death

The proportion of children dying in health facilities nearly doubled from 25.5% to 47.7% among 5–14-year-olds and increased from 25.0% to 42.1% among 15–19-year-olds in the first and final periods, respectively. This was similar to children aged 1–4 years (28.9% to 44.3% in first and final periods, respectively) (Figure 4 and Appendix 1 Table S8). Deaths due to communicable diseases more commonly occurred in health facilities than deaths due to non-communicable diseases and Injuries (Appendix 1 Figure S3). The place of death distribution by major causes of deaths was similar among 5–19-year-olds and 1–4-year-olds (Appendix 1 Figure S4).

## Discussion

Major changes in cause of death patterns and place of death have occurred among older children and adolescents in the rural and semi-urban population of Magu HDSS between 1995 and 2022, despite limited programmatic focus on this age group during the past decades.

In this population, communicable diseases contributed to the greatest share of mortality during 1995–2004 period, accounting for more than 70% of deaths at ages 5–19 years, with HIV/AIDS, respiratory tract illnesses, malaria and fever unspecified contributing more than half of these deaths (45.8%). Similar findings have been reported in other low- and lower-middle-income countries [10,28,33], where the largest share of cause of death in this age group was also due to communicable diseases.

Mortality in this population declined steadily during 1995–2022, and the major declines in 5–14 and 15–19 mortality were almost entirely driven by the decline in communicable disease mortality, including HIV/AIDS/TB mortality, respiratory illnesses mortality and mortality from other and unspecified communicable diseases. The reductions in HIV/AIDS/TB mortality have been observed in other adult mortality studies within the same population [34], availability of antiretroviral therapy in the latter periods [35] and the implementation of sexual and reproductive health interventions targeting younger people [36,37] may have an impact on this population.

Malaria mortality also declined, but its share in the cause of death distribution did not change much: 19% and 17% of deaths among 5–19-year-olds in 1995–2002 and 2015–2022, respectively. Magu HDSS area is located in a malaria-endemic region, which may explain why its share was higher than the global estimates. However, there may also be overdiagnosis of malaria as a cause of death or misclassification of other fever deaths as malaria death which is one of the limitations of the VA methodology, which tends to over-diagnose endemic causes of deaths, as reported elsewhere [10,33]. Further investigations are essential.

With a decline in communicable diseases, non-communicable diseases and injuries have gained relative importance among 5–19-year-olds, contributing more than 50% of deaths in this age group during the final period. This was similar to the findings of a recent large-scale verbal autopsy study which looked into community deaths in the Iringa region in southern Tanzania from 2018 to 2021, where non-communicable diseases and injuries contributed to 55.7% of deaths among 5–15-year-olds, compared to 37% among children aged 1–4 years [38]. This shift in mortality from infectious diseases to non-communicable diseases and injuries is striking and in line with epidemiological transition theory. Mortality due to sickle-cell disease, primarily based on the results of the physician reviews, was high and accounted for nearly 40% of all non-communicable disease deaths among 5–19-year-olds in Magu HDSS. This raises concerns about the communities’ awareness and health services responsiveness regarding this condition which is known to be common in the region surrounding Lake Victoria, with reports of a prevalence of sickle cell trait as high as 32%) [39]. A greater awareness in prevalent communities, early detection, and adequate management by health workers is essential for the improvement of the survival chances, and adequate management by health workers is essential for improving the chances of survival of children with sickle-cell disease.

While among injuries, road traffic accidents also accounted for up to 40% of all Injuries among 5–19-year-olds in the study area; the proportion of children and adolescents dying from road traffic accidents did not show much improvement between the second and final periods. Increased urbanization in the area leading into increased road use is likely to influence this shift, however, proper enforcement of traffic laws (including speed limits and seatbelts) and helmets for motorbikes are necessary, as well as improvement of road infrastructures in order to increase safety.

The analyses of disparities in cause-specific mortality showed that boys had higher mortality for all three major cause groups (communicable diseases, non-communicable diseases and injuries). The sex differences can be related to a mix of biological and behavioral causes, although beyond a higher risk of motor vehicle injuries [40], no immediate explanation emerges.

Children living in the poorest households had about two times higher mortality risks for communicable diseases, but not for the other cause groups, in both 5–14- and 15–19-year age groups. The observed changes in the cause distribution should, therefore, lead to a reduction of all-cause mortality inequalities, while targeting the poorest in communicable disease control efforts remains a priority. Individuals residing in rural areas had a significantly higher risk for non-communicable diseases among both 5–14- and 15–19-year-olds. These rural/urban differences are possibly associated with differential access to health facilities with appropriate diagnostic and curative services, although it is not clear why this is not occurring for communicable diseases. Stigma following certain illnesses such as sickle cell disease as reported elsewhere [41–43] may also be a contributing factor if it is more severe in rural compared to semi-urban villages. Further investigations are needed, and efforts to increase access to equitable health care and further integrate non-communicable disease prevention and treatment for children into health programming are critical.

Regarding the place of death, the proportion of deaths at 5–19 years occurring in health facilities increased over time to just above 40% in 2015–2022. This is higher than reported for several other populations where under 30% of deaths occurred in health facilities [11,38]. Nevertheless, most communicable diseases and non-communicable disease deaths still occurred outside health facilities, which emphasizes the usefulness of the VA system in capturing community deaths in the region. However, it also highlights the importance of addressing the knowledge gap surrounding many severe health conditions for individuals in rural communities.

The large decline in mortality due to communicable diseases among 5–19 years of age was similar to that observed at ages 1–4 years. This resulted in a smaller decline in all-cause mortality among children 5–19 years as other causes (non-communicable diseases and injuries) played a greater role and did not decline. This also implies that further declines among older children and adolescents require a much broader effort than at ages 1–4 years where further reductions in communicable diseases still have a large impact on all-cause mortality in that age group.

Even though the Magu HDSS is a localized population, it is considered typical for Tanzania’s population in terms of socio-economic and health characteristics [17]. For instance, its trends in all-cause mortality among children 5–19 years were close to the overall trend in Tanzania based on national surveys [18]; therefore, we expected similarities between cause of death distributions for Tanzania and the Magu HDSS. Our comparison with the global estimates for Tanzania showed indeed many similarities but also differences (e.g. malaria and injuries) which further stress the importance of local data to inform estimation models.

The limitations of this study include the imprecision of VA interviews, since VA is a crude instrument relying on information provided by a close family member and is subject to recall and other biases. As a result, we cannot rule out the possibility of over-or-under reporting of certain causes of death. The VA interviews were conducted shortly after a case of death occurred, reducing the risk of recall bias. Furthermore, VA questions were administered by well-trained medical personnel in order to minimize any potential cultural bias. Additionally, previous research has shown good validity of the Magu HDSS for the diagnosis of AIDS mortality in adults [44]. Second, the changes in the VA instruments over time may have affected our trend analysis, even though this was addressed by the standardization of each instrument to the ALPHA standard. Our analysis showed that the mortality due to non-communicable diseases and injuries was likely underestimated in the first period. Despite the relatively small number of deaths recorded and analyzed in the Magu HDSS, the use of three time periods presents a stable trend that provides a valuable benchmark for older children and adolescent mortality assessment in Tanzania. Further, our analysis of the determinants was only guided by the available data and previous literature among 5–19-year-olds. We did not have data on pathways in which these determinants (wealth, area of residence) may influence survival in this age group, as proposed in comprehensive frameworks of mortality determinants among younger children [45]. Therefore, we limited our study to describe the existing inequalities.

## Conclusion

Our study provides evidence of a major mortality transition among older children and adolescents during 1995–2022, driven entirely by a substantial decline in mortality due to communicable diseases which was remarkably similar in size to the decline at ages 1–4 years. This was accompanied by an increase in children 5–19 years dying in health facilities, though still 53% died at home. Further reductions in communicable disease mortality, especially malaria, will be essential for continued all-cause mortality declines, but increasingly programs will need to address non-communicable diseases, including sickle-cell anemia, and injuries. Tanzania’s health strategies have begun to include older children’s and adolescents’ health as a program area. Our unique assessment of mortality and causes of death among 5–19-year-olds based on 28 years of demographic and health surveillance provides the kind of data needed to guide such policies and programmes and help establish priority areas for public health action and health system strengthening.

## Supplementary Material

Appendix 1.docx

Appendix 2_ALPHA spec.docx

## Data Availability

Data used in the analysis reported in this manuscript can be accessed through a formal request to the corresponding author.
