# Supplementary material for: Causes of death among older children and adolescents (5–19 years) in the Magu Health and Demographic Surveillance Study, Tanzania, 1995–2022
Source: Glob Health Action. 2024 Nov 7;17(1):2425470. doi: 10.1080/16549716.2024.2425470 (PMC11544727; doi:10.1080/16549716.2024.2425470)
Supplement: Appendix 1.docx [file ZGHA_A_2425470_SM6266.docx]

**Appendix 1: Additional Tables and graphs**

**Table S1: Cause of death categories and broad cause of death groupings**

| **Specific Cause of death** | **Broad cause of death** | **WHO Classification** |
| --- | --- | --- |
| Sepsis/septicemia | Communicable diseases | Group 1: Communicable (infectious and parasitic diseases and maternal, perinatal and nutritional conditions) |
| Acute resp infect incl pneumonia |  |  |
| HIV/AIDS related death |  |  |
| Diarrhoeal diseases |  |  |
| Malaria |  |  |
| Measles |  |  |
| Meningitis and encephalitis |  |  |
| Tetanus |  |  |
| Pulmonary tuberculosis |  |  |
| Pertussis |  |  |
| Dengue fever |  |  |
| Other and unspecified infect disease |  |  |
| Severe malnutrition | Malnutrition |  |
| Ectopic pregnancy | Direct Obstetric Causes |  |
| Abortion-related death |  |  |
| Pregnancy-induced hypertension |  |  |
| Obstetric haemorrhage |  |  |
| Obstructed labour |  |  |
| Pregnancy-related sepsis |  |  |
| Anaemia in pregnancy |  |  |
| Ruptured uterus |  |  |
| Other and unspecified maternal CoD |  |  |
| Oral neoplasms | Non-Communicable disease | Group 2: Non-communicable (chronic) conditions |
| Digestive neoplasms |  |  |
| Respiratory neoplasms |  |  |
| Breast neoplasms |  |  |
| Reproductive neoplasms |  |  |
| Other and unspecified neoplasms |  |  |
| Severe anaemia |  |  |
| Diabetes mellitus |  |  |
| Acute cardiac disease |  |  |
| Stroke |  |  |
| Sickle cell disease |  |  |
| Other and unspecified cardiac disease |  |  |
| Asthma |  |  |
| Acute abdomen |  |  |
| Liver cirrhosis |  |  |
| Renal failure |  |  |
| Epilepsy |  |  |
| Other and unspecified non-communicable disease |  |  |
| Road traffic accident | Injuries | Group 3: Injuries |
| Other transport accident |  |  |
| Drowning |  |  |
| Accidental fall |  |  |
| Accidental expos to smoke fire & flame |  |  |
| Contact with venomous plant/animal |  |  |
| Poisoning |  |  |
| Intentional self-harm |  |  |
| Assault |  |  |
| Exposure to force of nature |  |  |
| Other and unspecified external CoD |  |  |
| Indeterminate | Indeterminate |  |

**
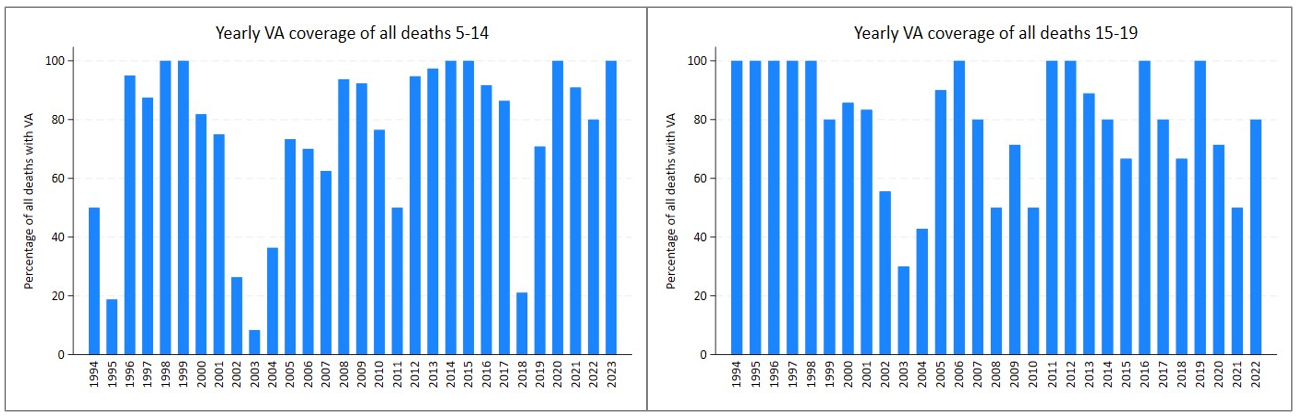
**

**Figure S1: Yearly VA coverage for 5-14- and 15–19-year-olds in Magu HDSS from 1995-2022**

**Table S2: VA coverage by background characteristics**

|  | **Number of deaths** | **Proportion of deaths with verbal autopsy (%)** |
| --- | --- | --- |
| **Overall** | 635 | 77.6 |
| **Sex**  Male  Female | 367  268 | 79.0  75.7 |
| **Age groups**  5-14  15-19 | 456  138 | 77.8  77.1 |
| **Calendar year**  1995-2004  2005-2014  2015-2022 | 226  233  176 | 67.3  83.3  83.5 |
| **Area of residence**  Semi-urban  Rural | 205  430 | 73.7  79.5 |
| **Wealth tertiles**  Poorest 33%  Middle  Richest 33%  Missing | 151  145  144  195 | 100.0  100.0  100.0  27.2 |

**Table S3: Number of deaths distribution by age, time period and broad causes among 5–19-year-olds in Magu HDSS (1995-2022)**

|  | **1995-2004** | **2005-2014** | **2015-2022** |
| --- | --- | --- | --- |
|  |  | **1-4 years** |  |
| **Total number of deaths** | 483 | 408 | 153 |
| **VA done** | 244 | 335 | 112 |
| **Indeterminate causes** | 25 | 19 | 5 |
| **Number with causes** | 219 | 316 | 107 |
| Communicable diseases | 184 (84.0) | 226 (71.5) | 75 (70.1) |
| Malnutrition | 7 (3.2) | 24 (7.6) | 3 (2.8) |
| Non-communicable diseases | 27 (12.3) | 39 (12.3) | 15 (14.0) |
| Injuries | 1 (0.5) | 27 (8.6) | 14 (13.1) |
|  |  |  |  |
|  | **5-14 years** | | |
| **Total number of deaths**  **VA done**  **Indeterminate causes** | 157  101  13 | 171  145  3 | 128  109  0 |
| **Number with causes** | 88 | 142 | 109 |
| Communicable diseases | 71 (80.7) | 73 (51.4) | 42 (38.5) |
| Malnutrition | 2 (2.3) | 7 (4.9) | 4 (3.7) |
| Direct obstetric causes | 1 (1.1) | 0 (0.0) | 0 (0.0) |
| Non-communicable diseases | 13 (14.8) | 42 (29.6) | 39 (35.8) |
| Injuries | 1 (1.1) | 20 (14.1) | 24 (22.0) |
|  |  |  |  |
|  | **15-19 years** | | |
| **Total number of deaths**  **VA done**  **Indeterminate causes**  **Number with causes** | 69  51  6  45 | 62  49  0  49 | 48  38  0  38 |
| Communicable diseases | 32 (71.1) | 19 (38.8) | 14 (36.8) |
| Malnutrition | 0 (0.0) | 0 (0.0) | 0 (0.0) |
| Direct obstetric causes | 6 (13.3) | 2 (4.1) | 2 (5.3) |
| Non-communicable diseases | 4 (8.9) | 17 (34.7) | 15 (39.5) |
| Injuries | 3 (6.7) | 11 (22.4) | 7 (18.4) |

**Table S4: Cause-specific mortality and rate of change by age and time period for broad causes among 5–19-year-olds in Magu HDSS (1995-2022)**

|  | **1995-2004** | **2005-2014** | **2015-2022** | **Rate of change (%) ***  **(1^st^ period-Final period)** |
| --- | --- | --- | --- | --- |
|  | **1-4** | | | |
| **Mortality probability per 10,000** | **546** | **396** | **160** | **-70.7** |
| Communicable diseases | 458.7 | 283.2 | 112.1 | -75.6 |
| Malnutrition | 17.5 | 30.1 | 4.5 | -74.3 |
| Non-communicable diseases | 67.3 | 48.9 | 22.4 | -66.7 |
| Injuries | 2.5 | 33.8 | 20.9 | 736.0 |
|  |  |  |  |  |
|  | **5-14** | | | |
| **Mortality probability per 10,000** | **235** | **191** | **135** | **-42.6** |
| Communicable diseases | 189.6 | 98.2 | 52.0 | -72.6 |
| Malnutrition | 5.3 | 9.4 | 5.0 | -7.2 |
| Direct obstetric causes | 2.7 | - | - | - |
| Non-communicable diseases | 34.7 | 56.5 | 48.3 | 39.1 |
| Injuries | 2.7 | 26.9 | 29.7 | 1013.1 |
|  |  |  |  |  |
|  | **15-19** | | | |
| **Mortality probability per 10,000** | **148** | **104** | **77** | **-48.0** |
| Communicable diseases | 105.2 | 40.3 | 28.4 | -73.0 |
| Malnutrition | - | - | - | - |
| Direct obstetric causes | 19.7 | 4.2 | 4.1 | -79.2 |
| Non-communicable diseases | 13.2 | 36.1 | 30.4 | 130.3 |
| Injuries | 9.9 | 23.3 | 14.2 | 43.4 |

***Rate of change=** $\left( \frac{\boldsymbol{Mortality at the end-Mortality at the beginning}}{\boldsymbol{Mortality at the beginning}} \right)\boldsymbol{\times100\%}$

**Table S5: Number of deaths distribution for specific causes by age and time periods among 5–19-year-olds in Magu HDSS (1995-2022)**

|  | **1995-2004**  **n (%)** | **2005-2014**  **n (%)** | **2015-2022**  **n (%)** |
| --- | --- | --- | --- |
| **Number of deaths** |  | **5-14 years** |  |
| **All** | **88** | **142** | **109** |
| HIV/TB | 9 (10.2) | 7 (5.0) | 4 (3.7) |
| Malaria and Fever unspecified | 21 (23.9) | 31 (21.8) | 22 (20.2) |
| Diarrhoeal diseases | 10 (11.4) | 8 (5.6) | 7 (6.4) |
| Respiratory tract illnesses | 9 (10.2) | 2 (1.4) | 0 (0.0) |
| Other and unspecified communicable diseases | 22 (25.0) | 25 (17.6) | 8 (7.3) |
| Malnutrition | 2 (2.3) | 7 (5.0) | 4 (3.7) |
| Direct obstetric causes | 1 (1.1) | 0 (0.0) | 0 (0.0) |
| Sickle cell disease | 4 (4.6) | 25 (17.6) | 18 (16.5) |
| Epilepsy | 6 (6.8) | 7 (5.0) | 5 (4.6) |
| Neoplasms | 0 (0.0) | 3 (2.1) | 3 (2.7) |
| Other non-communicable diseases | 3 (3.4) | 7 (4.9) | 13 (11.9) |
| Drowning | 1 (1.1) | 6 (4.2) | 8 (7.3) |
| Road traffic accident | 0 (0.0) | 9 (6.3) | 9 (8.3) |
| Other Injuries | 0 (0.0) | 5 (3.5) | 8 (7.3) |
|  | **15-19 years** | | |
| **All** | **45** | **49** | **38** |
| HIV/TB | 12 (26.7) | 8 (16.3) | 2 (5.3) |
| Malaria and Fever unspecified | 5 (11.1) | 3 (6.1) | 4 (10.5) |
| Diarrhoeal diseases | 2 (4.4) | 0 (0.0) | 3 (7.9) |
| Respiratory tract illnesses | 5 (11.1) | 0 (0.0) | 0 (0.0) |
| Other and unspecified communicable diseases | 8 (17.8) | 8 (16.3) | 5 (13.2) |
| Malnutrition | 0 (0.0) | 0 (0.0) | 0 (0.0) |
| Direct obstetric causes | 6 (16.3) | 2 (4.1) | 2 (5.3) |
| Sickle cell disease | 1 (2.2) | 2 (4.1) | 3 (7.9) |
| Epilepsy | 3 (6.7) | 4 (8.2) | 3 (7.9) |
| Neoplasms | 0 (0.0) | 6 (12.2) | 1 (2.6) |
| Other non-communicable diseases | 0 (0.0) | 5 (10.2) | 8 (21.0) |
| Drowning | 2 (4.4) | 1 (2.0) | 1 (2.6) |
| Road traffic accident  Other Injuries | 1 (2.2)  0 (0.0) | 5 (10.2)  5 (10.2) | 2 (5.3)  4 (10.5) |
|  |  | **Total** |  |
| **All** | **133** | **191** | **147** |
| HIV/TB | 21 (15.8) | 15 (7.8) | 6 (4.1) |
| Malaria and Fever unspecified | 26 (19.5) | 34 (17.8) | 26 (17.7) |
| Diarrhoeal diseases | 12 (9.0) | 8 (4.2) | 10 (6.8) |
| Respiratory tract illnesses | 14 (10.5) | 2 (1.0) | 0 (0.0) |
| Other and unspecified communicable diseases | 30 (22.6) | 33 (17.3) | 13 (8.8) |
| Malnutrition | 2 (1.5) | 7 (3.7) | 4 (2.7) |
| Direct obstetric causes | 7 (5.3) | 2 (1.0) | 2 (1.4) |
| Sickle cell disease | 5 (2.8) | 27 (14.1) | 21 (14.3) |
| Epilepsy | 9 (6.8) | 11 (5.8) | 8 (5.4) |
| Neoplasms | 0 (0.0) | 9 (4.7) | 4 (2.7) |
| Other non-communicable diseases | 3 (2.3) | 12 (6.3) | 21 (14.3) |

**Table S5 (continued…): Number of deaths distribution for specific causes by age and time periods among 5–19-year-olds in Magu HDSS (1995-2022)**

|  | **1995-2004**  **n (%)** | **2005-2014**  **n (%)** | **2015-2022**  **n (%)** |
| --- | --- | --- | --- |
| **Number of deaths** |  | **Total** |  |
| Drowning | 3 (2.3) | 7 (3.7) | 9 (6.1) |
| Road traffic accident  Other Injuries | 1 (0.7)  0 (0.0) | 14 (7.3)  10 (5.2) | 11 (7.5)  12 (8.2) |

**Table S6: Number of deaths distribution by age, sex and specific causes among 5–19-year-olds in Magu HDSS (1995-2022)**

|  | **5-14** | | **15-19** | | **Total** |
| --- | --- | --- | --- | --- | --- |
|  | **Male** | **Female** | **Male** | **Female** |  |
| **Number of deaths** | **n (%)** | **n (%)** | **n (%)** | **n (%)** | **n (%)** |
| All | 204 | 135 | 72 | 60 | 471 |
| HIV/TB | 9 (4.4) | 11 (8.2) | 12 (16.7) | 10 (16.7) | 42 (8.9) |
| Malaria | 32 (15.7) | 28 (20.7) | 4 (5.6) | 7 (11.6) | 71 (15.1) |
| Diarrhoeal diseases | 14 (6.9) | 11 (8.2) | 5 (6.9) | 0 (0.0) | 30 (6.4) |
| Respiratory tract illnesses | 6 (2.9) | 5 (3.7) | 4 (5.6) | 1 (1.7) | 16 (3.4) |
| Other communicable diseases | 45 (22.1) | 25 (18.5) | 15 (20.8) | 7 (11.7) | 92 (19.5) |
| Malnutrition | 6 (2.9) | 7 (5.2) | 0 (0.0) | 0 (0.0) | 13 (2.8) |
| Direct obstetric causes | - | 1 (0.8) | - | 10 (16.6) | 11 (2.3) |
| Sickle cell disease | 28 (13.7) | 19 (14.1) | 2 (2.8) | 4 (6.8) | 53 (11.3) |
| Epilepsy | 10 (4.9) | 8 (5.9) | 5 (6.9) | 5 (8.3) | 28 (5.9) |
| Neoplasms | 4 (2.0) | 2 (1.5) | 2 (2.8) | 5 (8.3) | 13 (2.8) |
| Other non-communicable diseases | 17 (8.3) | 6 (4.4) | 7 (9.7) | 6 (10.0) | 36 (7.6) |
| Drowning | 13 (6.4) | 2 (1.5) | 4 (5.6) | 0 (0.0) | 19 (4.0) |
| Road traffic accident | 11 (5.4) | 7 (5.2) | 6 (8.3) | 2 (3.3) | 26 (5.5) |
| Other Injuries | 9 (4.4) | 3 (2.2) | 6 (8.3) | 3 (5.0) | 21 (4.5) |

*Other communicable diseases- Other (Meningitis, Septicemia, unspecified communicable diseases)

**
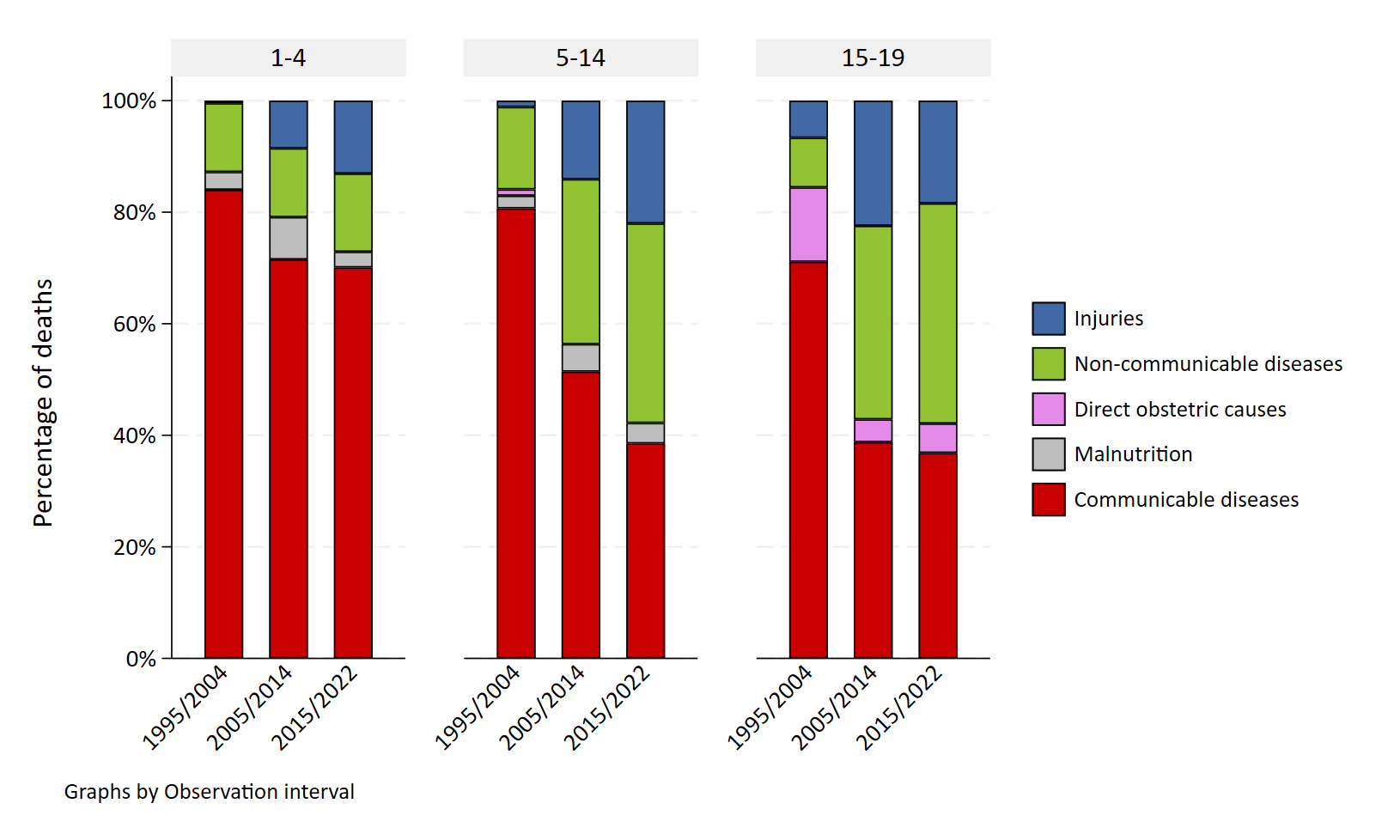
**

**Figure S2: Mortality fractions among 5–19-year-olds, compared to 1–4-year-olds in Magu HDSS (1995-2022)**

**Table S7: Comparison of cause of death distribution (in percentage) among 5–14-year-olds between Magu HDSS and global estimates for Tanzania from 2010 to 2022**

| **Specific cause of death** | **UN IGME (%)** | **Global burden of disease (%)** | **Magu HDSS (%)** |
| --- | --- | --- | --- |
| HIV/TB | 10.2 | 14.7 | 3.5 |
| Malaria | 9.9 | 4.5 | 22.6 |
| Diarrhoea | 8.3 | 13.6 | 6 |
| Respiratory tract illness | 8.6 | 9.7 | 1 |
| Other Communicable diseases | 19.5 | 9.8 | 13.6 |
| Malnutrition |  | 2.0 | 3.5 |
| Neoplasms | 4.5 | 5.2 | 2 |
| Other non-communicable diseases | 20.5 | 24.6 | 26.6 |
| Drowning | 6.3 | 3.3 | 7 |
| Road traffic accidents | 3.6 | 4.3 | 8.5 |
| Other Injuries | 8.7 | 8.1 | 5.5 |
| Total | 100 | 100 | 100 |

**Table S8: Changes in distribution (in percent) of place of death among 5–19-year-olds in Magu HDSS, 1995-2022**

| **Place of death** | **1-4** | | | **5-14** | | | **15-19** | | |
| --- | --- | --- | --- | --- | --- | --- | --- | --- | --- |
|  | **1995-2004**  **(n=242)** | **2005-2014**  **(n=333)** | **2015-2022**  **(n=124)** | **1995-2004**  **(n=98)** | **2005-2014**  **(n=145)** | **2015-2022**  **(n=109)** | **1995-2004**  **(n=40)** | **2005-2014**  **(n=49)** | **2015-2022**  **(n=38)** |
| **Hospital/Health centre** | 28.9 | 40.5 | 44.3 | 25.5 | 40.0 | 47.7 | 25.0 | 32.7 | 42.1 |
| **Home** | 51.7 | 47.5 | 46.0 | 59.2 | 39.3 | 29.4 | 60.0 | 36.7 | 44.7 |
| **Traditional healer** | 6.2 | 4.2 | 1.6 | 7.1 | 4.8 | 2.7 | 0.0 | 6.1 | 0.0 |
| **Other** | 13.2 | 7.8 | 8.1 | 8.2 | 15.9 | 20.2 | 15.0 | 24.5 | 13.2 |


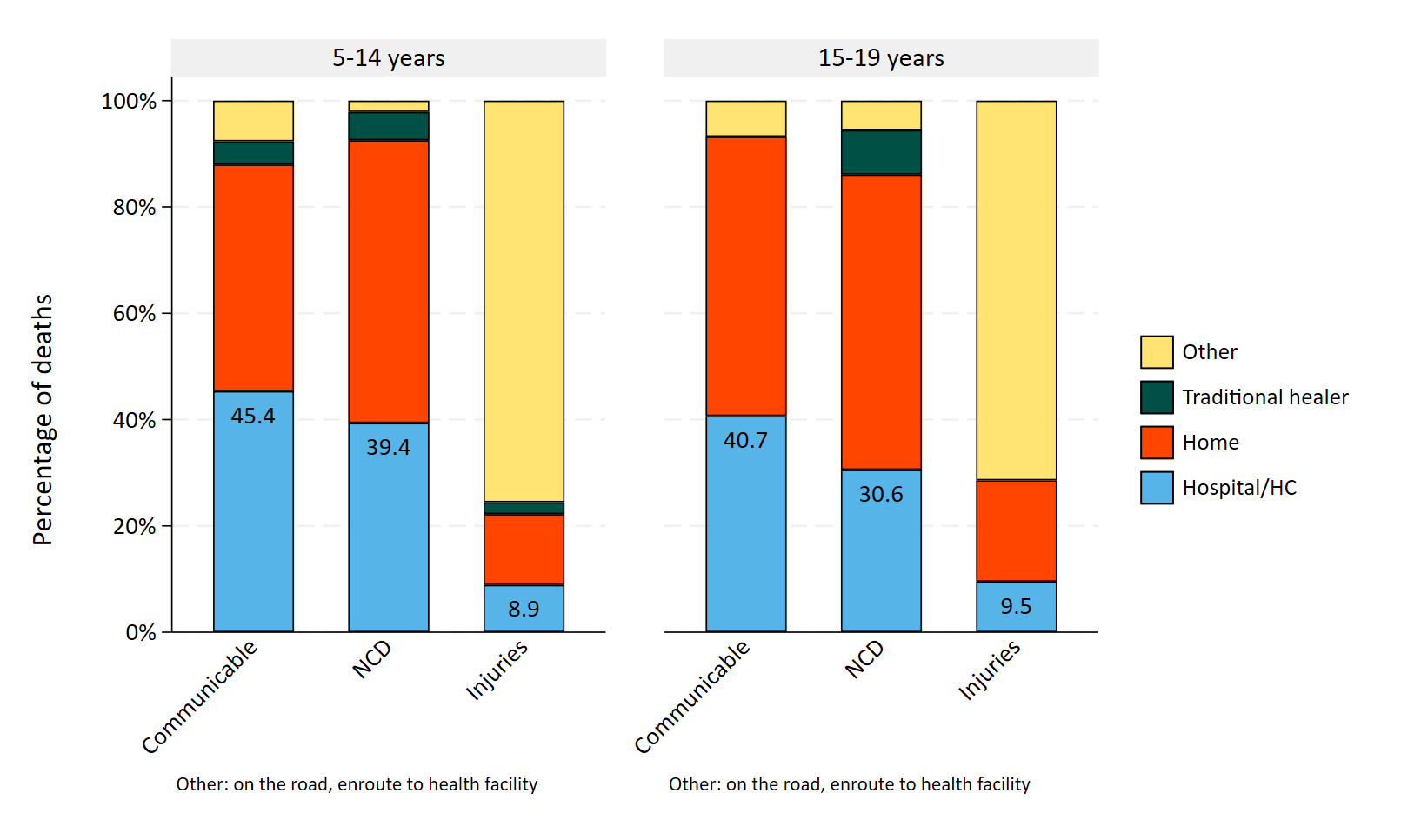


**Figure S3: Place of death distribution by major causes of deaths among 5–19-year-olds in Magu HDSS**

**
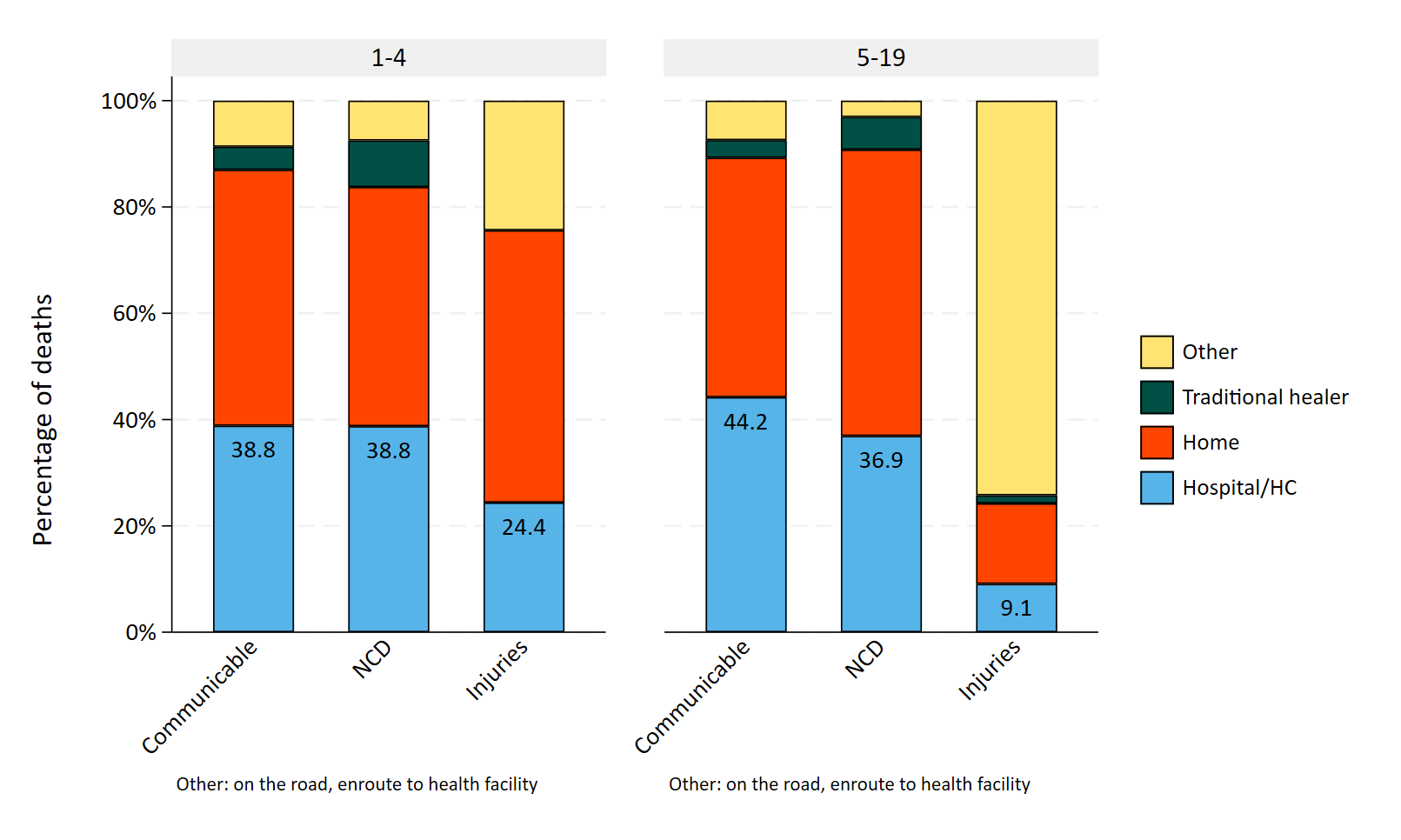
**

**Figure S4: Place of death distribution by major causes of deaths comparing 5–19-year-olds with 1–4-year-olds in Magu HDSS**

**Table S9: All-cause mortality probabilities for 5–19-year-olds by 9-year time periods and by sex**

| **Time period** | **5-9** | | **10-14** | | **15-19** | | **5-14** | |
| --- | --- | --- | --- | --- | --- | --- | --- | --- |
|  | **Male** | **Female** | **Male** | **Female** | **Male** | **Female** | **Male** | **Female** |
| 1995-2004 | 16.1 | 13.8 | 9.0 | 8.2 | 16.0 | 13.4 | 25.0 | 21.9 |
| 2005-2014 | 15.7 | 9.2 | 9.0 | 4.5 | 9.8 | 11.1 | 24.6 | 13.7 |
| 2015-2022 | 9.3 | 7.1 | 6.8 | 3.9 | 8.0 | 7.4 | 16.0 | 11.0 |

**Table S10: Socio-demographic risk factors of cause-specific mortality among 5–14-year-olds in Magu HDSS (Crude and Adjusted analysis)**

|  | **Communicable diseases** | | **Non-communicable diseases** | | **Injuries** | |
| --- | --- | --- | --- | --- | --- | --- |
|  | **Crude analysis**  **CHR (95% CI)** | **Adjusted analysis**^¥^  **AHR (95% CI)** | **Crude analysis**  **CHR (95% CI)** | **Adjusted analysis**^¥^  **AHR (95% CI)** | **Crude analysis**  **CHR (95% CI)** | **Adjusted analysis**^¥^  **AHR (95% CI)** |
| **Calendar time**  1995- 2004  2005-2014  2015-2022 | 1  0.77 (0.55, 1.08)  0.30 (0.20, 0.47) *** | 1  0.76 (0.53, 1.09)  0.30 (0.19, 0.48) *** | 1  2.21 (1.23, 3.96) **  1.67 (0.91, 3.05) | 1  2.34 (1.26, 4.34) **  1.71 (0.91, 3.24) | 1  4.44 (1.31, 15.07) *  4.00 (1.17, 13.66) * | 1  6.10 (1.42, 26.31) *  5.20 (1.20, 22.59) * |
| **Sex**  Male  Female | 1.41 (1.03, 1.91) *  1 | 1.43 (1.03, 1.98) *  1 | 1.60 (1.06, 2.41) *  1 | 1.62 (1.07, 2.45) *  1 | 2.18 (1.10, 4.31) *  1 | 2.38 (1.18, 4.82) *  1 |
| **Area of residence**  Semi-urban  Rural | 1  1.41 (1.02, 1.94) * | 1  0.92 (0.63, 1.37) | 1  1.91 (1.22, 3.00) ** | 1  1.80 (1.06, 3.04) * | 1  1.73 (0.86, 3.49) | 1  1.92 (0.83, 4.43) |
| **Wealth tertiles**  Poorest 33%  Middle  Richest 33% | 1.84 (1.21, 2.80) **  1.36 (0.86, 2.14)  1 | 1.80 (1.10, 2.95) *  1.33 (0.82, 2.16)  1 | 1.58 (0.93, 2.66)  1.39 (0.80, 2.43)  1 | 1.11 (0.61, 2.04)  1.11 (0.62, 2.01)  1 | 1.26 (057, 2.78)  0.98 (0.41, 2.30)  1 | 0.90 (0.36, 2.25)  0.80 (0.32, 1.99)  1 |

**p<0.05; **p<0.01; p<0.001; CHR = Crude Hazard ratio; AHR = Adjusted Hazard Ratio*

*^¥^Adjusted for calendar time, sex, area of residence and wealth tertiles*

**Table S11: Socio-demographic risk factors of cause-specific mortality among 15-19-year-olds in Magu HDSS (Crude and Adjusted analysis)**

|  | **Communicable diseases** | | **Non-communicable diseases** | | **Injuries** | |
| --- | --- | --- | --- | --- | --- | --- |
|  | **Crude analysis**  **CHR (95% CI)** | **Adjusted analysis**^¥^  **AHR (95% CI)** | **Crude analysis**  **CHR (95% CI)** | **Adjusted analysis**^¥^  **AHR (95% CI)** | **Crude analysis**  **CHR (95% CI)** | **Adjusted analysis**^¥^  **AHR (95% CI)** |
| **Calendar time**  1995- 2004  2005-2014  2015-2022 | 1  0.41 (0.22, 0.75) **  0.26 (0.13, 0.53) *** | 1  0.51 (0.27, 0.98) *  0.33 (0.16, 0.68) ** | 1  2.94 (0.98, 8.81)  1.37 (0.41, 4.54) | 1  2.76 (0.92, 8.25)  1.24 (0.37, 4.12) | 1  2.70 (0.75, 9.68)  1.39 (0.35, 5.54) | 1  2.27 (0.62, 8.25)  1.32 (0.33, 5.30) |
| **Sex**  Male  Female | 1.26 (0.74, 2.13)  1 | 1.12 (0.63, 1.99)  1 | 0.79 (0.38, 1.67)  1 | 0.76 (0.36, 1.59)  1 | 3.61 (1.21, 10.79) *  1 | 4.58 (1.33, 15.74) *  1 |
| **Area of residence**  Semi-urban  Rural | 1  1.44 (0.84, 2.49) | 1  0.73 (0.37, 1.46) | 1  2.85 (1.16, 7.03) * | 1  2.92 (1.05, 8.12) * | 1  0.79 (0.33, 1.89) | 1  0.72 (0.26, 1.97) |
| **Wealth tertiles**  Poorest 33%  Middle  Richest 33% | 1.89 (0.97, 3.69)  0.69 (0.29, 1.62)  1 | 2.12 (0.95, 4.71)  0.71 (0.29, 1.74)  1 | 1.70 (0.63, 4.60)  1.77 (0.65, 4.77)  1 | 0.90 (0.29, 2.78)  1.15 (0.39, 3.37)  1 | 1.20 (0.32, 4.47)  2.51 (0.79, 8.01)  1 | 1.38 (0.32, 5.99)  2.70 (0.80, 9.15)  1 |

**p<0.05; **p<0.01; p<0.001; CHR = Crude Hazard ratio; AHR = Adjusted Hazard Ratio*

*^¥^Adjusted for calendar time, sex, area of residence and wealth tertiles*
