# Supplementary material for: Causes of death among older children and adolescents (5–19 years) in the Magu Health and Demographic Surveillance Study, Tanzania, 1995–2022
Source: Glob Health Action. 2024 Nov 7;17(1):2425470. doi: 10.1080/16549716.2024.2425470 (PMC11544727; doi:10.1080/16549716.2024.2425470)
Supplement: Appendix 2_ALPHA spec.docx [file ZGHA_A_2425470_SM6265.docx]

**Appendix 2: ALPHA VA data specification**

Data specification: Verbal autopsy data for child deaths

Sites are asked to make the most detailed and complete version available from their verbal autopsy data.

NOTE: Data should only come from the verbal autopsy questionnaire, do not populate or clean it using data from other sources.

**Verbal Autopsy file – one record for each child death with VA (child = age at death 1 month -> 12 years). Variables highlighted in light orange rows are variables that will only be included if you have used the WHO 2016 questionnaire.**

| **Variable name** | | **Description** | **Coding** | **Notes** |
| --- | --- | --- | --- | --- |
| **idno** | | Person ID number | site specific | Numeric IDs long integer format, unique for an individual |
| **idno_interviewer** | | ID number for the interviewer | site specific | This ID number should enable us to identify which VAs were done by the same interviewer |
| **responder_type** | | Relationship of respondent to the deceased (i.e. parent, not related) | site specific (please include labels) |  |
| **study_name** | | Name of your study field site | site specific | Character – please be consistent across data sets |
| **va_interview_date** | | Date of VA interview | in Stata format |  |
| **va_date_of_death** | | Reported date of death | in Stata format |  |
| **va_age_at_death** | | Age at death in years | 1-11 as reported  88 Refused  99 Don’t know  999 less than 1 years old | Code as stated in VA interview – consistency checks with DSS date of birth will be performed in do-file |
| **va_months_at_death** | | Age at death in months | 1-11 as reported  88 Refused  99 Don’t know  999 not applicable (older than 1 year) | Code as stated in VA interview – consistency checks with DSS date of birth will be performed in do-file |
| **va_sex** | | Male or female | 1 Male  2 Female  88 Refused  99 Don’t know | Code as stated in VA interview – consistency checks with DSS sex will be performed in do-file |
| **va_die_where** | | Where did the deceased die? | 0 Hospital  1 Health Centre  2 Home  3 En route to facility  4 Other  88 Refused  99 Don’t know |  |
| **va_final_ill** | | Did final illness last at least 3 weeks? | 0 No  1 Yes  88 Refused  99 Don’t know |  |
| **va_ill_day** | For how long was s/he ill before death (days) | 0-31 as reported  88 Refused  99 Don’t know | Less than 24 hours = 0 days |  |
| **va_ill_month** | For how long was s/he ill before death (months) | 1-60 as reported  88 Refused  99 Don’t know |  |  |
| **va_ill_year** | For how long was s/he ill before death (years) | 1-11 as reported  88 Refused  99 Don’t know |  |  |
| **va_ill_age_month** | How old was the child when the fatal illness started? (months) | 1-11 as reported  88 Refused  99 Don’t know |  |  |
| **va_ill_age_year** | How old was the child when the fatal illness started? (years) | 1-11 as reported  88 Refused  99 Don’t know |  |  |
| **va_sudden** | Was death very sudden or unexpected | 0 No  1 Yes  88 Refused  99 Don’t know |  |  |
| **va_season** | Season of death | 0 Dry  1 wet  88 Refused  99 Don’t know |  |  |
| **va_heart_dis** | Any medical diagnosis of heart disease | 0 No  1 Yes  88 Refused  99 Don’t know |  |  |
| **va_tuber** | Any medical diagnosis of TB | 0 No  1 Yes  88 Refused  99 Don’t know |  |  |
| **va_hiv** | Was an HIV test ever positive | 0 No  1 Yes  88 Refused  99 Don’t know |  |  |
| **va_aids** | Was there any diagnosis by a health professional of AIDS | 0 No  1 Yes  88 Refused  99 Don’t know |  |  |
| **va_hypert** | Any medical diagnosis of high blood pressure | 0 No  1 Yes  88 Refused  99 Don’t know |  |  |
| **va_diabetes** | Any medical diagnosis of diabetes | 0 No  1 Yes  88 Refused  99 Don’t know |  |  |
| **va_asthma** | Any medical diagnosis of asthma | 0 No  1 Yes  88 Refused  99 Don’t know |  |  |
| **va_epilepsy** | Any medical diagnosis of epilepsy | 0 No  1 Yes  88 Refused  99 Don’t know |  |  |
| **va_cancer** | Any medical diagnosis of cancer | 0 No  1 Yes  88 Refused  99 Don’t know |  |  |
| **va_stroke** | Any medical diagnosis of stroke | 0 No  1 Yes  88 Refused  99 Don’t know |  |  |
| **va_sickle** | Any medical diagnosis of haemoglobinopathy | 0 No  1 Yes  88 Refused  99 Don’t know |  |  |
| **va_kidney_dis** | Any medical diagnosis of kidney disease | 0 No  1 Yes  88 Refused  99 Don’t know |  |  |
| **va_liver_dis** | Any medical diagnosis of liver disease | 0 No  1 Yes  88 Refused  99 Don’t know |  |  |
| **va_measles** | Any medical diagnosis of measles | 0 No  1 Yes  88 Refused  99 Don’t know |  |  |
| **va_malaria_pos** | Recent positive malaria test | 0 No  1 Yes  88 Refused  99 Don’t know |  |  |
| **va_malaria_neg** | Recent negative malaria test | 0 No  1 Yes  88 Refused  99 Don’t know |  |  |
| **va_dengue** | Any medical diagnosis of dengue fever | 0 No  1 Yes  88 Refused  99 Don’t know |  |  |
| **va_fever** | Any fever | 0 No  1 Yes  88 Refused  99 Don’t know |  |  |
| **va_fever_day** | How long did the fever last (days) | 0-31 as reported  88 Refused  99 Don’t know | Less than 24 hours = 0 days |  |
| **va_fever_month** | How long did the fever last (months) | 1-60 as reported  88 Refused  99 Don’t know |  |  |
| **va_fever_die** | Did the fever continue until death | 0 No  1 Yes  88 Refused  99 Don’t know |  |  |
| **va_fever_sev** | How severe was the fever? | 0 Mild  1 Moderate  2 Severe  88 Refused  99 Don’t know |  |  |
| **va_fever_patt** | What was the pattern of fever | 0 Continuous  1 On and off  2 Only at night  88 Refused  99 Don’t know |  |  |
| **va_night_sw** | Any excessive night sweats | 0 No  1 Yes  88 Refused  99 Don’t know |  |  |
| **va_cough** | Any cough | 0 No  1 Yes  88 Refused  99 Don’t know |  |  |
| **va_cough_day** | How long did the cough last (days) | 0-31 as reported  88 Refused  99 Don’t know | Less than 24 hours = 0 days |  |
| **va_cough_month** | How long did the cough last (months) | 1-60 as reported  88 Refused  99 Don’t know |  |  |
| **va_cough_pr** | Any productive cough, with sputum | 0 No  1 Yes  88 Refused  99 Don’t know |  |  |
| **va_cough_sev** | Was the cough very severe? | 0 No  1 Yes  88 Refused  99 Don’t know |  |  |
| **va_bl_cough** | Any coughing with blood | 0 No  1 Yes  88 Refused  99 Don’t know |  |  |
| **va_whoop** | Any distinctive whoop (associated with characteristic whooping sound of pertussis) | 0 No  1 Yes  88 Refused  99 Don’t know |  |  |
| **va_diff_br** | Any breathing problem? | 0 No  1 Yes  88 Refused  99 Don’t know |  |  |
| **va_diff_br_day** | How long did the difficult breathing last (days) | 0-31 as reported  88 Refused  99 Don’t know |  |  |
| **va_diff_br_month** | How long did the difficult breathing last (months) | 1-60 as reported  88 Refused  99 Don’t know |  |  |
| **va_diff_br_year** | How long did the difficult breathing last (years) | 1-11 as reported  88 Refused  99 Don’t know |  |  |
| **va_diff_br_patt** | Was the difficulty breathing continuous or on and off | 0 Continuous  1 On and off  88 Refused  99 Don’t know |  |  |
| **va_rapid_br** | Any rapid breathing | 0 No  1 Yes  88 Refused  99 Don’t know |  |  |
| **va_rapid_br_day** | How long did the rapid breathing last (days) | 0-31 as reported  88 Refused  99 Don’t know |  |  |
| **va_rapid_br_month** | How long did the rapid breathing last (months) | 1-60 as reported  88 Refused  99 Don’t know |  |  |
| **va_breathless** | Any breathlessness | 0 No  1 Yes  88 Refused  99 Don’t know |  |  |
| **va_breathless_day** | How long did the breathlessness last (days) | 0-31 as reported  88 Refused  99 Don’t know | Less than 24 hours = 0 days |  |
| **va_breathless_month** | How long did the breathlessness last (months) | 1-60 as reported  88 Refused  99 Don’t know |  |  |
| **va_exert_br** | Any breathlessness on exertion | 0 No  1 Yes  88 Refused  99 Don’t know |  |  |
| **va_lying_br** | Any breathlessness lying flat | 0 No  1 Yes  88 Refused  99 Don’t know |  |  |
| **va_chest_in** | Any chest indrawing | 0 No  1 Yes  88 Refused  99 Don’t know |  |  |
| **va_wheeze** | Any wheezing | 0 No  1 Yes  88 Refused  99 Don’t know |  |  |
| **va_grunt** | Any grunting | 0 No  1 Yes  88 Refused  99 Don’t know |  |  |
| **va_stridor** | Any stridor | 0 No  1 Yes  88 Refused  99 Don’t know |  |  |
| **va_ch_pain** | Any chest pain | 0 No  1 Yes  88 Refused  99 Don’t know |  |  |
| **va_ch_pain_day** | How many days before death did s/he have chest pain | Days as reported  88 Refused  99 Don’t know | Less than 24 hours = 0 days |  |
| **va_yellow** | Any yellow discolouration of the eyes | 0 No  1 Yes  88 Refused  99 Don’t know |  |  |
| **va_yellow_day** | How did s/he have yellow discolouration of the eyes? (days) | 0-31 as reported  88 Refused  99 Don’t know | Less than 24 hours = 0 days |  |
| **va_yellow_month** | How did s/he have yellow discolouration of the eyes? (months) | 1-60 as reported  88 Refused  99 Don’t know |  |  |
| **va_diarr** | Any diarrhoea | 0 No  1 Yes  88 Refused  99 Don’t know |  |  |
| **va_diarr_day** | How long did the diarrhoea last (days) | 0-31 as reported  88 Refused  99 Don’t know | Less than 24 hours = 0 days |  |
| **va_diarr_month** | How long did the diarrhoea last (months) | 1-60 as reported  88 Refused  99 Don’t know |  |  |
| **va_diarr_num** | How many stools did the baby or child have on the day that loose liquid stools were most frequent? | Enter number of stools  88 Refused  99 Don’t know |  |  |
| **va_diarr_die_d** | How long before death did the diarrhoea start (days) | 0-31 as reported  88 Refused  99 Don’t know |  |  |
| **va_diarr_die_m** | How long before death did the diarrhoea start (months) | 1-60 as reported  88 Refused  99 Don’t know |  |  |
| **va_diarr_die** | Did the diarrhoea continue until death | 0 No  1 Yes  88 Refused  99 Don’t know |  |  |
| **va_bl_diarr** | Any diarrhoea with blood | 0 No  1 Yes  88 Refused  99 Don’t know |  |  |
| **va_bl_diarr_die** | Was there blood in the stool up until death | 0 No  1 Yes  88 Refused  99 Don’t know |  |  |
| **va_vomiting** | Any vomiting | 0 No  1 Yes  88 Refused  99 Don’t know |  |  |
| **va_vomit_die** | Did s/he vomit in the week preceding the death | 0 No  1 Yes  88 Refused  99 Don’t know |  |  |
| **va_bl_vomit** | Any vomiting with blood or “coffee grounds” | 0 No  1 Yes  88 Refused  99 Don’t know |  |  |
| **va_black_vom** | Was the vomit black? | 0 No  1 Yes  88 Refused  99 Don’t know |  |  |
| **va_abd_prob** | Any abdominal problem | 0 No  1 Yes  88 Refused  99 Don’t know |  |  |
| **va_abd_pain** | Any abdominal pain | 0 No  1 Yes  88 Refused  99 Don’t know |  |  |
| **va_abd_pain_hour** | How long did the abdominal pain last (hours) | 0-23 as reported  88 Refused  99 Don’t know |  |  |
| **va_abd_pain_day** | How long did the abdominal pain last (days) | 1-31 as reported  88 Refused  99 Don’t know |  |  |
| **va_abd_pain_month** | How long did the abdominal pain last (months) | 1-60 as reported  88 Refused  99 Don’t know |  |  |
| **va_abd_pain_sev** | Was the abdominal pain severe? | 0 No  1 Yes  88 Refused  99 Don’t know |  |  |
| **va_abd_pain_where** | Was the pain in the upper or lower belly abdomen | 0 Upper  1 Lower  2 Upper & lower  88 Refused  99 Don’t know |  |  |
| **va_swe_abd** | Any more than usually protruding abdomen | 0 No  1 Yes  88 Refused  99 Don’t know |  |  |
| **va_swe_abd_day** | How long before death did s/he have protruding abdomen (days) | 0-31 as reported  88 Refused  99 Don’t know | Less than 24 hours = 0 days |  |
| **va_swe_abd_month** | How long before death did s/he have protruding abdomen (months) | 1-60 as reported  88 Refused  99 Don’t know |  |  |
| **va_swe_abd_rapid** | How rapidly did s/he develop the protruding abdomen? | 0 Rapidly  1 Slowly  88 Refused  99 Don’t know |  |  |
| **va_mass_abd** | Any lump inside the abdomen | 0 No  1 Yes  88 Refused  99 Don’t know |  |  |
| **va_mass_abd_day** | How long did s/he have a mass in the abdomen (days) | 0-31 as reported  88 Refused  99 Don’t know | Less than 24 hours = 0 days |  |
| **va_mass_abd_month** | How long did s/he have a mass in the abdomen (months) | 1-60 as reported  88 Refused  99 Don’t know |  |  |
| **va_headache** | Any severe headache | 0 No  1 Yes  88 Refused  99 Don’t know |  |  |
| **va_skin** | Any skin problems | 0 No  1 Yes  88 Refused  99 Don’t know |  |  |
| **va_skin_red** | Did s/he have areas of the skin with redness and swelling? | 0 No  1 Yes  88 Refused  99 Don’t know |  |  |
| **va_ulc_any** | Any ulcers or sores? | 0 No  1 Yes  88 Refused  99 Don’t know |  |  |
| **va_ulc_pus** | Did the sores have clear fluid or pus? | 0 No  1 Yes  88 Refused  99 Don’t know |  |  |
| **va_ulc_feet** | Any ulcers/ abscesses or sores on the feet | 0 No  1 Yes  88 Refused  99 Don’t know |  |  |
| **va_pus_feet** | Did the ulcer on the foot ooze pus? | 0 No  1 Yes  88 Refused  99 Don’t know |  |  |
| **va_pus_feet_day** | How long did the ulcer on the foot ooze pus (days) | 0-31 as reported  88 Refused  99 Don’t know | Less than 24 hours = 0 days |  |
| **va_pus_feet_month** | How long did the ulcer on the foot ooze pus (months) | 1-60 as reported  88 Refused  99 Don’t know |  |  |
| **va_ulc_oth** | Any ulcers/ abscesses or sores on body, apart from feet | 0 No  1 Yes  88 Refused  99 Don’t know |  |  |
| **va_rash** | Any rash | 0 No  1 Yes  88 Refused  99 Don’t know |  |  |
| **va_rash_day** | For how many days did she have skin rash? | Number of days as reported  88 Refused  99 Don’t know | Less than 24 hours = 0 days |  |
| **va_rash_face** | Was there rash on the face? | 0 No  1 Yes  88 Refused  99 Don’t know |  |  |
| **va_rash_trunk** | Was there rash on the trunk/abdomen? | 0 No  1 Yes  88 Refused  99 Don’t know |  |  |
| **va_rash_extremities** | Was there rash on the extremities? | 0 No  1 Yes  88 Refused  99 Don’t know |  |  |
| **va_rash_all** | Was there rash everywhere? | 0 No  1 Yes  88 Refused  99 Don’t know |  |  |
| **va_measrash** | Any measles rash | 0 No  1 Yes  88 Refused  99 Don’t know |  |  |
| **va_skin_flake** | Did her/his skin flake off in patches? | 0 No  1 Yes  88 Refused  99 Don’t know |  |  |
| **va_skin_black** | Did her/his skin have areas that turned black? | 0 No  1 Yes  88 Refused  99 Don’t know |  |  |
| **va_herpes** | Any herpes zoster | 0 No  1 Yes  88 Refused  99 Don’t know |  |  |
| **va_stiff_neck** | Any stiff neck | 0 No  1 Yes  88 Refused  99 Don’t know |  |  |
| **va_stiff_neck_day** | How long before death did s/he have a stiff neck (days) | 0-31 as reported  88 Refused  99 Don’t know | Less than 24 hours = 0 days |  |
| **va_stiff_neck_month** | How long before death did s/he have a stiff neck (months) | 1-60 as reported  88 Refused  99 Don’t know |  |  |
| **va_pain_neck** | Did s/he have a painful neck during the illness that led to death? | 0 No  1 Yes  88 Refused  99 Don’t know |  |  |
| **va_pain_neck_day** | How long before death did s/he have a painful neck (days) | 0-31 as reported  88 Refused  99 Don’t know | Less than 24 hours = 0 days |  |
| **va_pain_neck_month** | How long before death did s/he have a painful neck (months) | 1-60 as reported  88 Refused  99 Don’t know |  |  |
| **va_any_coma** | Was s/he unconscious during the illness that led to death? | 0 No  1 Yes  88 Refused  99 Don’t know |  |  |
| **va_coma** | Was there a coma > 24hrs | 0 No  1 Yes  88 Refused  99 Don’t know |  |  |
| **va_coma_hour** | How long before death did unconsciousness start (hour) | 0-23 as reported  88 Refused  99 Don’t know | Less than 1 hour = 0 hours |  |
| **va_coma_day** | How long before death did unconsciousness start (day) | 1-99 as reported  888 Refused  999 Don’t know | If more than 99 days reported, then enter 99 |  |
| **va_coma_sudden** | Did the coma come on suddenly (at least within a single day) | 0 No  1 Yes  88 Refused  99 Don’t know |  |  |
| **va_coma_die** | Did the unconsciousness continue until death? | 0 No  1 Yes  88 Refused  99 Don’t know |  |  |
| **va_convul** | Any convulsions or fits | 0 No  1 Yes  88 Refused  99 Don’t know |  |  |
| **va_convul_gen** | Did s/he experience any generalised convulsions or fits during the illness that led to death? | 0 No  1 Yes  88 Refused  99 Don’t know |  |  |
| **va_convul_min** | How many minutes did the convulsions last? | Minutes as reported  888 Refused  999 Don’t know | Less than 1 minute = 0 minutes |  |
| **va_convul_coma** | Became unconscious immediately after convulsions | 0 No  1 Yes  88 Refused  99 Don’t know |  |  |
| **va_conv_d1** | Did the baby have convulsions starting within the first day of life? | 0 No  1 Yes  88 Refused  99 Don’t know | Only for children <1 years old |  |
| **va_conv_d2** | Did the baby have convulsions starting on the second day or later after birth? | 0 No  1 Yes  88 Refused  99 Don’t know | Only for children <1 years old |  |
| **va_arch_b** | Did the baby’s body become stiff, with the back arched backwards? | 0 No  1 Yes  88 Refused  99 Don’t know | Only for children <1 years old |  |
| **va_urine** | Any abnormality of urine | 0 No  1 Yes  88 Refused  99 Don’t know |  |  |
| **va_uri_ret** | Any urinary retention | 0 No  1 Yes  88 Refused  99 Don’t know |  |  |
| **va_exc_urine** | Any excessive urination | 0 No  1 Yes  88 Refused  99 Don’t know |  |  |
| **va_uri_haem** | Any haematuria | 0 No  1 Yes  88 Refused  99 Don’t know |  |  |
| **va_wt_loss** | Any weight loss | 0 No  1 Yes  88 Refused  99 Don’t know |  |  |
| **va_wasting** | Any severe wasting | 0 No  1 Yes  88 Refused  99 Don’t know |  |  |
| **va_or_cand** | Any oral candidiasis | 0 No  1 Yes  88 Refused  99 Don’t know |  |  |
| **va_rigidity** | Any rigidity/lockjaw | 0 No  1 Yes  88 Refused  99 Don’t know |  |  |
| **va_lump** | Any localised lump or lesion | 0 No  1 Yes  88 Refused  99 Don’t know |  |  |
| **va_lump_mouth** | Any lump or lesion in mouth | 0 No  1 Yes  88 Refused  99 Don’t know |  |  |
| **va_lump_armpit** | Any lump or lesion in armpit | 0 No  1 Yes  88 Refused  99 Don’t know |  |  |
| **va_lump_neck** | Any lumps/swelling in neck | 0 No  1 Yes  88 Refused  99 Don’t know |  |  |
| **va_lump_gen** | Any lump or lesion in groin or genitals | 0 No  1 Yes  88 Refused  99 Don’t know |  |  |
| **va_swe_face** | Any facial swelling/ puffiness | 0 No  1 Yes  88 Refused  99 Don’t know |  |  |
| **va_swe_face_day** | How long did s/he have puffiness of the face (days) | 0-31 as reported  88 Refused  99 Don’t know | Less than 24 hours = 0 days |  |
| **va_swe_face_month** | How long did s/he have puffiness of the face (months) | 1-60 as reported  88 Refused  99 Don’t know |  |  |
| **va_swe_leg** | Any swollen legs or feet? | 0 No  1 Yes  88 Refused  99 Don’t know |  |  |
| **va_swe_leg_day** | How long did s/he have swelling of legs (days) | 0-31 as reported  88 Refused  99 Don’t know | Less than 24 hours = 0 days |  |
| **va_swe_leg_month** | How long did s/he have swelling of legs (months) | 1-60 as reported  88 Refused  99 Don’t know |  |  |
| **va_swe_ankles** | Any swelling of both feet/ankles | 0 No  1 Yes  88 Refused  99 Don’t know |  |  |
| **va_swe_all** | Did s/he have general puffiness all over his/her body? | 0 No  1 Yes  88 Refused  99 Don’t know |  |  |
| **va_anaemia** | Any anaemia/paleness | 0 No  1 Yes  88 Refused  99 Don’t know |  |  |
| **va_exc_drink** | Any excessive water intake | 0 No  1 Yes  88 Refused  99 Don’t know |  |  |
| **va_hair** | Any abnormal hair colouring | 0 No  1 Yes  88 Refused  99 Don’t know |  |  |
| **va_eye_sunk** | Were eyes sunken | 0 No  1 Yes  88 Refused  99 Don’t know |  |  |
| **va_paralysis** | Was there paralysis | 0 No paralysis  1 One side  2 Both sides  88 Refused  99 Don’t know |  |  |
| **va_para_right** | Was the right side paralysed? | 0 No  1 Yes  88 Refused  99 Don’t know |  |  |
| **va_para_left** | Was the left side paralysed? | 0 No  1 Yes  88 Refused  99 Don’t know |  |  |
| **va_para_lower** | Was the lower side of the body paralysed? | 0 No  1 Yes  88 Refused  99 Don’t know |  |  |
| **va_para_upper** | Was the upper side of the body paralysed? | 0 No  1 Yes  88 Refused  99 Don’t know |  |  |
| **va_para_leg** | Was one leg paralysed? | 0 No  1 Yes  88 Refused  99 Don’t know |  |  |
| **va_para_arm** | Was one arm paralysed? | 0 No  1 Yes  88 Refused  99 Don’t know |  |  |
| **va_para_body** | Was the whole body paralysed? | 0 No  1 Yes  88 Refused  99 Don’t know |  |  |
| **va_bleed_any** | Did s/he bleed from anywhere? | 0 No  1 Yes  88 Refused  99 Don’t know |  |  |
| **va_bleed** | Was the any bleeding from mouth, nose and anus | 0 No  1 Yes  88 Refused  99 Don’t know |  |  |
| **va_menstrual** | Was there any bleeding between menstrual periods (women aged 12-50 only) | 0 No  1 Yes  88 Refused  99 Don’t know | (Left in case some child questionnaires extend to age 15) |  |
| **va_drink_diff** | Any difficulty or pain in swallowing | 0 No  1 Yes  88 Refused  99 Don’t know |  |  |
| **va_drink_diff_day** | How long before death did s/he have difficulty swallowing (days) | 0-31 as reported  88 Refused  99 Don’t know | Less than 24 hours = 0 days |  |
| **va_drink_diff_month** | How long before death did s/he have difficulty swallowing (months) | 1-60 as reported  88 Refused  99 Don’t know |  |  |
| **va_drink_diff_type** | Was the difficulty with swallowing with solids, liquids or both? | 0 Solids  1 Liquids  2 Both  88 Refused  99 Don’t know |  |  |
| **va_pain_swallow** | Did s/he have pain upon swallowing? | 0 No  1 Yes  88 Refused  99 Don’t know |  |  |
| **va_fed_d1** | Was the baby able to suckle or bottle-feed within the first 24 hours after birth? | 0 No  1 Yes  88 Refused  99 Don’t know | Only for children <1 years old |  |
| **va_suckle** | Did the baby ever suckle in a normal way? | 0 No  1 Yes  88 Refused  99 Don’t know | Only for children <1 years old |  |
| **va_nosuckle** | Did the baby stop suckling? | 0 No  1 Yes  88 Refused  99 Don’t know | Only for children <1 years old |  |
| **va_nosuckle_day** | How many days after birth did the baby stop suckling? | 0-31 as reported  88 Refused  99 Don’t know | Only for children <1 years old  Less than 24 hours = 0 days |  |
| **va_nosuckle_month** | How many months after birth did the baby stop suckling? | 1-11 as reported  88 Refused  99 Don’t know | Only for children <1 years old |  |
| **va_born_month** | How many months long was the pregnancy before the child was born? | 0-9 as stated  88 Refused  99 Don’t know |  |  |
| **va_born_early** | Had the pregnancy lasted less than 34 weeks when the baby was born? | 0 No  1 Yes  88 Refused  99 Don’t know |  |  |
| **va_born_3437** | Had the pregnancy lasted between 34 and 37 weeks when the baby was born? | 0 No  1 Yes  88 Refused  99 Don’t know |  |  |
| **va_born_38** | Had the pregnancy lasted more than 37 weeks when the baby was born? | 0 No  1 Yes  88 Refused  99 Don’t know |  |  |
| **va_ab_size** | Was the baby of abnormal size? | 0 No  1 Yes  88 Refused  99 Don’t know |  |  |
| **va_born_small** | Was the baby smaller than normal, weighing under 2.5kgs? | 0 No  1 Yes  88 Refused  99 Don’t know | Only for children <1 years old |  |
| **va_born_vsmall** | Was the baby very much smaller than normal, weighing under 1kg? | 0 No  1 Yes  88 Refused  99 Don’t know | Only for children <1 years old |  |
| **va_born_big** | Was the baby larger than normal, weighing more than 4.5kgs? | 0 No  1 Yes  88 Refused  99 Don’t know | Only for children <1 years old |  |
| **va_baby_weight** | What was the weight (in grams) of the baby at birth? | 0-6000 as stated  7777 greater than 6000grams  8888 Refused  9999 Don’t know |  |  |
| **va_twin** | Was the child part of multiple birth? | 0 No  1 Yes  88 Refused  99 Don’t know | Only for children <1 years old |  |
| **va_birth_order** | Was the child the first, second or later in the birth order | 0 First  1 Second or later  88 Refused  99 Don’t know | Only for children <1 years old |  |
| **va_compreg** | Was there any complications in the late part of the pregnancy (defined as the last 3 months, before labour) | 0 No  1 Yes  88 Refused  99 Don’t know |  |  |
| **va_comdel** | Was the child born in a complicated delivery? | 0 No  1 Yes  88 Refused  99 Don’t know |  |  |
| **va_font_hi** | Did the child have a bulging or raised fontanelle | 0 No  1 Yes  88 Refused  99 Don’t know | Only for children <18 months |  |
| **va_font_lo** | Did the child have a sunken fontanelle | 0 No  1 Yes  88 Refused  99 Don’t know | Only for children <18 months |  |
| **va_unw_d1** | Did the baby become unresponsive or unconscious soon after birth (within less than 24 hours)? | 0 No  1 Yes  88 Refused  99 Don’t know | Only for children <1 years old |  |
| **va_unw_d2** | Did the baby become unresponsive or unconscious more than 1 day after birth? | 0 No  1 Yes  88 Refused  99 Don’t know | Only for children <1 years old |  |
| **va_devel** | Was the baby/the child growing normally? | 0 No  1 Yes  88 Refused  99 Don’t know |  |  |
| **va_born_malf** | Did the baby/the child have any malformation? For example, body part too large or too small, additional growth on body. | 0 No  1 Yes  88 Refused  99 Don’t know | Only for children <1 years old |  |
| **va_mlf_bk** | Did the baby/the child have a swelling/defect on the back? | 0 No  1 Yes  88 Refused  99 Don’t know | Only for children <1 years old |  |
| **va_mlf_lh** | Did the baby/child have a very large head? | 0 No  1 Yes  88 Refused  99 Don’t know | Only for children <1 years old |  |
| **va_mlf_sh** | Did the baby/child have a very small head? | 0 No  1 Yes  88 Refused  99 Don’t know | Only for children <1 years old |  |
| **va_b_num** | How many births, including stillbirths, did the baby’s mother have before this baby? | Number as reported  88 Refused  99 Don’t know | Only for children <1 years old |  |
| **va_hivtest** | Has the baby’s mother even been tested for HIV? | 0 No  1 Yes  88 Refused  99 Don’t know |  |  |
| **va_mhiv** | Has the baby’s mother ever been told she has HIV/AIDS by a health worker? | 0 No  1 Yes  88 Refused  99 Don’t know |  |  |
| **va_malive** | Is the mother still alive? | 0 No  1 Yes  88 Refused  99 Don’t know | Only for children <1 years old |  |
| **va_mdie** | Did the mother die before, during or after the delivery? | 0 Before delivery  1 During delivery  2 After delivery  88 Refused  99 Don’t know | Only for children <1 years old |  |
| **va_mdie_day** | How long after the delivery did the mother die? (days) | 0-6 as reported  88 More than 1 year  99 Don’t know | If less than 1 day (24 hours) then enter 0 days.  Only for children <1 years old |  |
| **va_mdie_week** | How long after the delivery did the mother die? (weeks) | 1-7 as reported  88 More than 1 year  99 Don’t know | Only for children <1 years old |  |
| **va_mdie_month** | How long after the delivery did the mother die? (months) | 2-60 as reported  88 More than 1 year  99 Don’t know | Only for children <1 years old |  |
| **va_b_delivery_type** | How was the baby delivered? | 0 normal vaginal delivery, no instruments  1 vaginal delivery with forceps / Ventuse  2 delivery by Caesarean section  88 Refused  99 Don’t know | Only for children <1 years old |  |
| **va_b_where_deliver** | Where did delivery take place | 0 At home  1 In transit  2 At health facility (undefined)  3 Hospital  4 Health centre | Only for children <1 years old |  |
| **va_b_bprof** | Had professional assistance at delivery | 0 No  1 Yes  88 Refused  99 Don’t know | Only for children <1 years old |  |
| **va_injury** | Any obvious recent injury | 0 No  1 Yes  88 Refused  99 Don’t know |  |  |
| **va_transport_road** | Was s/he in a road transport accident | 0 No  1 Yes  88 Refused  99 Don’t know |  |  |
| **va_transport_role** | What was her/his role in the road traffic accident? | 0 Pedestrian  1 Passenger in car or light vehicle  2 Passenger in bus or heavy vehicle  3 Passenger on motorcycle  4 Passenger on pedal cycle  5 Other  88 Refused  99 Don’t know |  |  |
| **va_transport_counter** | What was the counterpart that was hit during the road traffic accident? | 0 Pedestrian  1 Stationary object  2 Car or light vehicle  3 Bus or heavy vehicle  4 Motorcycle  5 Pedal cycle  6 Other  88 Refused  99 Don’t know |  |  |
| **va_transport_oth** | Was s/he in a non-road transport accident | 0 No  1 Yes  88 Refused  99 Don’t know |  |  |
| **va_fall** | Had s/he fallen recently | 0 No  1 Yes  88 Refused  99 Don’t know |  |  |
| **va_drowning** | Did s/he drown | 0 No  1 Yes  88 Refused  99 Don’t know |  |  |
| **va_burn** | Was s/he burnt by heat, steam or fire | 0 No  1 Yes  88 Refused  99 Don’t know |  |  |
| **va_assault** | Injured in some kind of violence or assault by another person | 0 No  1 Yes  88 Refused  99 Don’t know |  |  |
| **va_firearm** | Was s/he injured by a firearm? | 0 No  1 Yes  88 Refused  99 Don’t know |  |  |
| **va_stab** | Was s/he stabbed, cut or pierced? | 0 No  1 Yes  88 Refused  99 Don’t know |  |  |
| **va_strangle** | Was s/he strangled? | 0 No  1 Yes  88 Refused  99 Don’t know |  |  |
| **va_blunt** | Was s/he injured by a blunt force? | 0 No  1 Yes  88 Refused  99 Don’t know |  |  |
| **va_venom** | Any poisoning, bite, sting from a venomous animal or insect | 0 No  1 Yes  88 Refused  99 Don’t know |  |  |
| **va_nonvenom** | Any bite or sting from a non-venomous animal or insect | 0 No  1 Yes  88 Refused  99 Don’t know |  |  |
| **va_animal** | What was the animal/insect | 0 Dog  1 Snake  2 Insect or scorpion  3 Other  88 Refused  99 Don’t know |  |  |
| **va_nature** | Was s/he injured by a force of nature | 0 No  1 Yes  88 Refused  99 Don’t know |  |  |
| **va_electrocution** | Was it electrocution? | 0 No  1 Yes  88 Refused  99 Don’t know |  |  |
| **va_poison** | Was there any poisoning? | 0 No  1 Yes  88 Refused  99 Don’t know |  |  |
| **va_inj_other** | Did s/he encounter any other injury? | 0 No  1 Yes  88 Refused  99 Don’t know |  |  |
| **va_inj_accident** | Was the injury accidental? | 0 No  1 Yes  88 Refused  99 Don’t know |  |  |
| **va_inj_intent** | Was h/she intentionally injured by another person or people | 0 No  1 Yes  88 Refused  99 Don’t know |  |  |
| **va_suicide** | Any suggestion of suicide | 0 No  1 Yes  88 Refused  99 Don’t know | (Left in case some child questionnaires extend to age 15) |  |
| **va_alcohol** | Was s/he known to drink alcohol | 0 No  1 Yes  88 Refused  99 Don’t know | (Left in case some child questionnaires extend to age 15) |  |
| **va_smoking** | Was s/he a known smoker | 0 No  1 Yes  88 Refused  99 Don’t know | (Left in case some child questionnaires extend to age 15) |  |
| **va_married** | Was she married/partnered at death | 0 No  1 Yes  88 Refused  99 Don’t know | (Left in case some child questionnaires extend to age 15) |  |
| **va_vaccin** | Was s/he adequately vaccinated | 0 No  1 Yes  88 Refused  99 Don’t know | - " - |  |
| **va_vacc_card** | Did the interviewer see the child’s vaccination card? | 0 No  1 Yes |  |  |
| **va_vacc_bcg** | Was BCG vaccine done? | 0 No  1 Yes  88 Refused  99 Don’t know |  |  |
| **va_vacc_dbt** | Was DBT vaccine done? | 0 No  1 Yes  88 Refused  99 Don’t know |  |  |
| **va_vacc_hepb** | Was Hep B done | 0 No  1 Yes  88 Refused  99 Don’t know |  |  |
| **va_vacc_hib** | Was HIB done | 0 No  1 Yes  88 Refused  99 Don’t know |  |  |
| **va_vacc_men** | Was Meningitis done | 0 No  1 Yes  88 Refused  99 Don’t know |  |  |
| **va_vacc_penta** | Was Penta done | 0 No  1 Yes  88 Refused  99 Don’t know |  |  |
| **va_vacc_pneumo** | Was Pneumo done? | 0 No  1 Yes  88 Refused  99 Don’t know |  |  |
| **va_vacc_polio** | Was Polio done? | 0 No  1 Yes  88 Refused  99 Don’t know |  |  |
| **va_vacc_rota** | Was Rota done? | 0 No  1 Yes  88 Refused  99 Don’t know |  |  |
| **va_no_vacc** | Were no vaccines reported on vaccination card? | 0 No  1 Yes  88 Refused  99 Don’t know |  |  |
| **va_out** | Was care sought outside the home while s/he has the illness? | 1 Yes  0 No  88 Refused  99 Don’t know |  |  |
| **va_out_trad** | Sought care from traditional healer | 0 No  1 Yes  88 Refused  99 Don’t know |  |  |
| **va_out_homeo** | Sought care from homeopath | 0 No  1 Yes  88 Refused  99 Don’t know |  |  |
| **va_out_relig** | Sought care from religious leader | 0 No  1 Yes  88 Refused  99 Don’t know |  |  |
| **va_out_hosp** | Sought care from government hospital | 0 No  1 Yes  88 Refused  99 Don’t know |  |  |
| **va_out_hc** | Sought care from government health centre | 0 No  1 Yes  88 Refused  99 Don’t know |  |  |
| **va_out_priv** | Sought care from private hospital | 0 No  1 Yes  88 Refused  99 Don’t know |  |  |
| **va_out_chw** | Sought care from community based practitioner associated with health system | 0 No  1 Yes  88 Refused  99 Don’t know |  |  |
| **va_out_tba** | Sought care from traditional birth attendant | 0 No  1 Yes  88 Refused  99 Don’t know |  |  |
| **va_out_privp** | Sought care from private physician | 0 No  1 Yes  88 Refused  99 Don’t know |  |  |
| **va_out_friend** | Sought care from friend/relative | 0 No  1 Yes  88 Refused  99 Don’t know |  |  |
| **va_out_pharm** | Sought care from pharmacy | 0 No  1 Yes  88 Refused  99 Don’t know |  |  |
| **va_treatment** | Treatment for final illness from a health facility | 0 No  1 Yes  88 Refused  99 Don’t know |  |  |
| **va_rehydrat** | Was oral rehydration required during final illness | 0 No  1 Yes  88 Refused  99 Don’t know |  |  |
| **va_iv** | Was an IV drip required during final illness | 0 No  1 Yes  88 Refused  99 Don’t know |  |  |
| **va_blood_tr** | Was blood transfusion required during final illness | 0 No  1 Yes  88 Refused  99 Don’t know |  |  |
| **va_nose** | Was treatment/food required through nose during final illness | 0 No  1 Yes  88 Refused  99 Don’t know |  |  |
| **va_antib_i** | Was antibiotic injection required during final illness | 0 No  1 Yes  88 Refused  99 Don’t know |  |  |
| **va_art** | Did s/he receive or need antiretroviral therapy? | 0 No  1 Yes  88 Refused  99 Don’t know |  |  |
| **va_surgery** | Any operation for the illness? | 0 No  1 Yes  88 Refused  99 Don’t know |  |  |
| **va_operation** | Was there an operation within one month of death | 0 No  1 Yes  88 Refused  99 Don’t know |  |  |
| **va_disch** | Was discharged from hospital very ill | 0 No  1 Yes  88 Refused  99 Don’t know |  |  |
| **va_shospf** | In the final days before death, did (s)he travel to the hospital or health facility? | 0 No  1 Yes  88 Refused  99 Don’t know |  |  |
| **va_strans** | Did they use motorised transport to get to the hospital or health facility? | 0 No  1 Yes  88 Refused  99 Don’t know |  |  |
| **va_sadmit** | Were there any problems during admission to the hospital or health facility? | 0 No  1 Yes  88 Refused  99 Don’t know |  |  |
| **va_streat** | Were there any problems with the way they were treated (medical treatment, procedures, inter-personal attitudes, respect, dignity) in the hospital or health facility? | 0 No  1 Yes  88 Refused  99 Don’t know |  |  |
| **va_smedic** | Were there any problems getting medications, or diagnostic tests in the hospital or health facility? | 0 No  1 Yes  88 Refused  99 Don’t know |  |  |
| **va_smore2** | Does it take more than 2 hours to get to the nearest hospital or health facility from the deceased’s household? | 0 No  1 Yes  88 Refused  99 Don’t know |  |  |
| **va_sdoubt** | In the final days before death, were there any doubts about whether medical care was needed? | 0 No  1 Yes  88 Refused  99 Don’t know |  |  |
| **va_stradm** | In the final days before death, was traditional medicine used? | 0 No  1 Yes  88 Refused  99 Don’t know |  |  |
| **va_smobph** | In the final days before death, did anyone use a telephone or cell phone to call for help? | 0 No  1 Yes  88 Refused  99 Don’t know |  |  |
| **va_scosts** | Over the course of the illness, did the total costs of care and treatment prohibit other household payments? | 0 No  1 Yes  88 Refused  99 Don’t know |  |  |
| **Informant’s understanding of deceased HIV service use** | | |  |  |
| **va_went_to_htc** | Did the deceased ever receive testing and counselling for HIV? | 0 No  1 Yes  88 Refused  99 Don’t know |  |  |
| **va_last_test_result** | What was the last HIV test result of the deceased | 0 negative  1 positive  88 Refused  99 Don’t know |  |  |
| **va_last_hiv_test_when** | When did deceased have last HIV test? | 1 in last year  2 more than a year ago  3 Never  88 Refused  99 Don’t know |  |  |
| **va_last_hiv_test_year** | When did deceased have last HIV test? | Year 4 digits  9999 Unknown |  |  |
| **va_last_hiv_test_month** | When did deceased have last HIV test? | Month as integer 1 to 12  88 Refused  99 Don’t know |  |  |
| **va_referred_art_ever** |  | 0 No  1 Yes  88 Refused  99 Don’t know |  |  |
| **va_referred_art_when** |  | 1 in last year  2 more than a year ago  88 Refused  99 Don’t know |  |  |
| **va_referred_art_year** |  | Year 4 digits  8888 Refused  9999 Don’t know |  |  |
| **va_referred_art_month** |  | Month as integer 1 to 12  88 Refused  99 Don’t know |  |  |
| **va_hivclinic_ever** | Whether deceased ever went to clinic/other service for HIV positive people | 0 No  1 Yes  88 Refused  99 Don’t know |  |  |
| **va_hivclinic_first_when** | When deceased first went to HIV clinic/service | 1 in last year  2 more than a year ago  3 Never  88 Refused  99 Don’t know |  |  |
| **va_hivclinic_first_year** |  | Year 4 digits  8888 Refused  9999 Don’t know |  |  |
| **va_hivclinic_first_month** |  | Month as integer 1 to 12  88 Refused  99 Don’t know |  |  |
| **va_hivclinic_current** | Was deceased attending HIV clinic/service around the time of death, were they still a patient. | 0 No  1 Yes  88 Refused  99 Don’t know |  |  |
| **va_assessed_for_art** | Was deceased ever assessed for ART treatment need? | 0 No  1 Yes  88 Refused  99 Don’t know |  |  |
| **va_first_assessed_when** |  | 1 in last year  2 more than a year ago  3 Never  88 Refused  99 Don’t know |  |  |
| **va_first_assessed_year** |  | Year 4 digits  8888 Refused  9999 Don’t know |  |  |
| **va_first_assessed_month** |  | Month as integer 1 to 12  88 Refused  99 Don’t know |  |  |
| **va_ever_prescribed_art** | Was the deceased ever prescribed ART treatment? | 0 No  1 Yes  88 Refused  99 Don’t know |  |  |
| **va_started_art** | Did deceased start ART treatment? | 0 No  1 Yes  88 Refused  99 Don’t know |  |  |
| **va_date_start_art** | When did the deceased start on ART treatment? | Date in Stata format |  |  |
| **va_art_current** | Was the deceased still on ART treatment when they died? | 0 No  1 Yes  88 Refused  99 Don’t know |  |  |
| **va_date_stop_art** | When did the deceased stop ART treatment? | Date in Stata format |  |  |
| **va_where_art** | Where did the deceased receive ART treatment? | 0 not treated  1 local clinic  2 clinic outside study area  88 Refused  99 Don’t know |  |  |

Data specification: Verbal autopsy data for adults

Sites are asked to make the most detailed and complete version available from their verbal autopsy data.

NOTE: Data should only come from the verbal autopsy questionnaire, do not populate or clean it using data from other sources.

**Verbal Autopsy file – one record for each adult death with VA (adults = age at death 12+ years).**

**Variables highlighted in light orange rows are variables that will only be included if you have used the WHO 2016 questionnaire.**

**Variables highlighted in green include information that should be available in older versions of the questionnaire, but have been added to the ALPHA data specification.**

| **Variable name** | | **Description** | **Coding** | **Notes** |
| --- | --- | --- | --- | --- |
| **idno** | | Person ID number | site specific | Numeric IDs long integer format, unique for an individual |
| **idno_interviewer** | | ID number for the interviewer | site specific | This ID number should enable us to identify which VAs were done by the same interviewer |
| **responder_type** | | Relationship of respondent to the deceased (i.e. parent, child, not related) | site specific (please include labels) |  |
| **study_name** | | Name of your study field site | site specific | Character – please be consistent across data sets |
| **va_interview_date** | | Date of VA interview | in Stata format |  |
| **va_date_of_death** | | Reported date of death | in Stata format |  |
| **va_age_at_death** | | Age at death in years | 12-89 as reported  90 = 90+  99 not stated | Code as stated in VA interview – consistency checks with DSS date of birth will be performed in do-file |
| **va_sex** | | Male or female | 1 Male  2 Female  88 Refused  99 Don’t know | Code as stated in VA interview – consistency checks with DSS sex will be performed in do-file |
| **va_die_where** | | Where did the deceased die? | 0 Hospital  1 Health Centre  2 Home  3 En route to facility  4 Other  88 Refused  99 Don’t know |  |
| **va_ill_day** | | For how long was s/he ill before death (days) | 0-31 as reported  88 Refused  99 Don’t know | Less than 24 hours = 0 days |
| **va_ill_month** | | For how long was s/he ill before death (months) | 1-60 as reported  88 Refused  99 Don’t know |  |
| **va_ill_year** | | For how long was s/he ill before death (years) | 1-11 as reported  88 Refused  99 Don’t know |  |
| **va_final_ill** | | Did final illness last at least 3 weeks? | 0 No  1 Yes  88 Refused  99 Don’t know |  |
| **va_sudden** | Was death very sudden or unexpected? (Suddenly means within 24 hours of being in regular health) | 0 No  1 Yes  88 Refused  99 Don’t know |  |  |
| **va_season** | Season of death | 0 Dry  1 wet  88 Refused  99 Don’t know |  |  |
| **va_heart_dis** | Any medical diagnosis of heart disease | 0 No  1 Yes  88 Refused  99 Don’t know |  |  |
| **va_tuber** | Any medical diagnosis of TB | 0 No  1 Yes  88 Refused  99 Don’t know |  |  |
| **va_hiv** | Was an HIV test ever positive | 0 No  1 Yes  88 Refused  99 Don’t know |  |  |
| **va_aids** | Was there any diagnosis by a health professional of AIDS | 0 No  1 Yes  88 Refused  99 Don’t know |  |  |
| **va_hypert** | Any medical diagnosis of high blood pressure | 0 No  1 Yes  88 Refused  99 Don’t know |  |  |
| **va_diabetes** | Any medical diagnosis of diabetes | 0 No  1 Yes  88 Refused  99 Don’t know |  |  |
| **va_asthma** | Any medical diagnosis of asthma | 0 No  1 Yes  88 Refused  99 Don’t know |  |  |
| **va_epilepsy** | Any medical diagnosis of epilepsy | 0 No  1 Yes  88 Refused  99 Don’t know |  |  |
| **va_cancer** | Any medical diagnosis of cancer | 0 No  1 Yes  88 Refused  99 Don’t know |  |  |
| **va_copd** | Any medical diagnosis of chronic obstructive pulmonary disease | 0 No  1 Yes  88 Refused  99 Don’t know |  |  |
| **va_dementia** | Any medical diagnosis of dementia | 0 No  1 Yes  88 Refused  99 Don’t know |  |  |
| **va_depress** | Any medical diagnosis of depression | 0 No  1 Yes  88 Refused  99 Don’t know |  |  |
| **va_stroke** | Any medical diagnosis of stroke | 0 No  1 Yes  88 Refused  99 Don’t know |  |  |
| **va_sickle** | Any medical diagnosis of haemoglobinopathy | 0 No  1 Yes  88 Refused  99 Don’t know |  |  |
| **va_kidney_dis** | Any medical diagnosis of kidney disease | 0 No  1 Yes  88 Refused  99 Don’t know |  |  |
| **va_liver_dis** | Any medical diagnosis of liver disease | 0 No  1 Yes  88 Refused  99 Don’t know |  |  |
| **va_measles** | Any medical diagnosis of measles | 0 No  1 Yes  88 Refused  99 Don’t know |  |  |
| **va_confusion** | Any medical diagnosis of memory loss or mental confusions | 0 No  1 Yes  88 Refused  99 Don’t know |  |  |
| **va_confuse_3** | Did the symptoms of mental confusion last 3 months or more? | 0 No  1 Yes  88 Refused  99 Don’t know |  |  |
| **va_confuse_day** | How long did the mental confusion last (days) | 0-31 as reported  88 Refused  99 Don’t know | Less than 24 hours = 0 days |  |
| **va_confuse_month** | How long did the mental confusion last (months) | 1-60 as reported  88 Refused  99 Don’t know |  |  |
| **va_malaria_pos** | Positive malaria test within one week of death | 0 No  1 Yes  88 Refused  99 Don’t know |  |  |
| **va_malaria_neg** | Negative malaria test within one week of death | 0 No  1 Yes  88 Refused  99 Don’t know |  |  |
| **va_dengue** | Any medical diagnosis of dengue fever | 0 No  1 Yes  88 Refused  99 Don’t know |  |  |
| **va_fever** | Any fever | 0 No  1 Yes  88 Refused  99 Don’t know |  |  |
| **va_fever_day** | How long did the fever last (days) | 0-31 as reported  88 Refused  99 Don’t know | Less than 24 hours = 0 days |  |
| **va_fever_month** | How long did the fever last (months) | 1-60 as reported  88 Refused  99 Don’t know |  |  |
| **va_fever_die** | Did the fever continue until death | 0 No  1 Yes  88 Refused  99 Don’t know |  |  |
| **va_fever_sev** | How severe was the fever? | 0 Mild  1 Moderate  2 Severe  88 Refused  99 Don’t know |  |  |
| **va_fever_patt** | What was the pattern of fever | 0 Continuous  1 On and off  2 Only at night  88 Refused  99 Don’t know |  |  |
| **va_night_sw** | Any excessive night sweats | 0 No  1 Yes  88 Refused  99 Don’t know |  |  |
| **va_cough** | Any cough | 0 No  1 Yes  88 Refused  99 Don’t know |  |  |
| **va_cough_day** | How long did the cough last (days) | 0-31 as reported  88 Refused  99 Don’t know | Less than 24 hours = 0 days |  |
| **va_cough_month** | How long did the cough last (months) | 1-60 as reported  88 Refused  99 Don’t know |  |  |
| **va_cough_pr** | Any productive cough | 0 No  1 Yes  88 Refused  99 Don’t know |  |  |
| **va_cough_sev** | Was the cough very severe? | 0 No  1 Yes  88 Refused  99 Don’t know |  |  |
| **va_bl_cough** | Any coughing with blood | 0 No  1 Yes  88 Refused  99 Don’t know |  |  |
| **va_diff_br** | Any breathing problem? | 0 No  1 Yes  88 Refused  99 Don’t know |  |  |
| **va_diff_br_day** | How long did the difficult breathing last (days) | 0-31 as reported  88 Refused  99 Don’t know |  |  |
| **va_diff_br_month** | How long did the difficult breathing last (months) | 1-60 as reported  88 Refused  99 Don’t know |  |  |
| **va_diff_br_year** | How long did the difficult breathing last (years) | 1-100 as reported  888 Refused  999 Don’t know |  |  |
| **va_diff_br_patt** | Was the difficulty breathing continuous or on and off | 0 Continuous  1 On and off  88 Refused  99 Don’t know |  |  |
| **va_rapid_br** | Any rapid breathing | 0 No  1 Yes  88 Refused  99 Don’t know |  |  |
| **va_rapid_br_day** | How long did the rapid breathing last (days) | 0-31 as reported  88 Refused  99 Don’t know |  |  |
| **va_rapid_br_month** | How long did the rapid breathing last (months) | 1-60 as reported  88 Refused  99 Don’t know |  |  |
| **va_breathless** | Any breathlessness | 0 No  1 Yes  88 Refused  99 Don’t know |  |  |
| **va_breathless_day** | How long did the breathlessness last (days) | 0-31 as reported  88 Refused  99 Don’t know | Less than 24 hours = 0 days |  |
| **va_breathless_month** | How long did the breathlessness last (months) | 1-60 as reported  88 Refused  99 Don’t know |  |  |
| **va_exert_br** | Any breathlessness on exertion | 0 No  1 Yes  88 Refused  99 Don’t know |  |  |
| **va_lying_br** | Any breathlessness lying flat | 0 No  1 Yes  88 Refused  99 Don’t know |  |  |
| **va_wheeze** | Any wheezing | 0 No  1 Yes  88 Refused  99 Don’t know |  |  |
| **va_ch_pain** | Any chest pain | 0 No  1 Yes  88 Refused  99 Don’t know |  |  |
| **va_ch_pain_sev** | Was the chest pain severe? | 0 No  1 Yes  88 Refused  99 Don’t know |  |  |
| **va_ch _day_die** | How many days before death did s/he have chest pain | Days as reported  88 Refused  99 Don’t know | Less than 24 hours = 0 days |  |
| **va_ch_pain_min** | How long did the chest pain last? (minutes) | 0-59 as reported  88 Refused  99 Don’t know |  |  |
| **va_ch_pain_hour** | How long did the chest pain last? (hours) | 1-23 as reported  88 Refused  99 Don’t know |  |  |
| **va_ch_pain_day** | How long did the chest pain last? (days) | 1-30 as reported  88 Refused  99 Don’t know |  |  |
| **va_yellow** | Any yellow discolouration of the eyes | 0 No  1 Yes  88 Refused  99 Don’t know |  |  |
| **va_yellow_day** | How did s/he have yellow discolouration of the eyes? (days) | 0-31 as reported  88 Refused  99 Don’t know | Less than 24 hours = 0 days |  |
| **va_yellow_month** | How did s/he have yellow discolouration of the eyes? (months) | 1-60 as reported  88 Refused  99 Don’t know |  |  |
| **va_diarr** | Any diarrhoea | 0 No  1 Yes  88 Refused  99 Don’t know |  |  |
| **va_diarr_day** | How long did the diarrhoea last (days) | 0-31 as reported  88 Refused  99 Don’t know | Less than 24 hours = 0 days |  |
| **va_diarr_month** | How long did the diarrhoea last (months) | 1-60 as reported  88 Refused  99 Don’t know |  |  |
| **va_bl_diarr** | Any diarrhoea with blood | 0 No  1 Yes  88 Refused  99 Don’t know |  |  |
| **va_bl_diarr_die** | Was there blood in the stool up until death | 0 No  1 Yes  88 Refused  99 Don’t know |  |  |
| **va_vomiting** | Any vomiting | 0 No  1 Yes  88 Refused  99 Don’t know |  |  |
| **va_vomit_die** | Did s/he vomit in the week preceding the death | 0 No  1 Yes  88 Refused  99 Don’t know |  |  |
| **va_vomit_day** | For how long before death did s/he vomit? (days) | 0-31 as reported  88 Refused  99 Don’t know | Less than 24 hours = 0 days |  |
| **va_vomit_month** | For how long before death did s/he vomit? (months) | 1-60 as reported  88 Refused  99 Don’t know |  |  |
| **va_bl_vomit** | Any vomiting with blood or “coffee grounds” | 0 No  1 Yes  88 Refused  99 Don’t know |  |  |
| **va_black_vom** | Was the vomit black? | 0 No  1 Yes  88 Refused  99 Don’t know |  |  |
| **va_abd_prob** | Any abdominal problem | 0 No  1 Yes  88 Refused  99 Don’t know |  |  |
| **va_abd_pain** | Any abdominal pain | 0 No  1 Yes  88 Refused  99 Don’t know |  |  |
| **va_abd_pain_hour** | How long did the abdominal pain last (hours) | 0-23 as reported  88 Refused  99 Don’t know |  |  |
| **va_abd_pain_day** | How long did the abdominal pain last (days) | 1-31 as reported  88 Refused  99 Don’t know |  |  |
| **va_abd_pain_month** | How long did the abdominal pain last (months) | 1-60 as reported  88 Refused  99 Don’t know |  |  |
| **va_abd_pain_sev** | Was the abdominal pain severe? | 0 No  1 Yes  88 Refused  99 Don’t know |  |  |
| **va_abd_pain_where** | Was the pain in the upper or lower belly abdomen | 0 Upper  1 Lower  2 Upper & lower  88 Refused  99 Don’t know |  |  |
| **va_swe_abd** | Any more than usually protruding abdomen | 0 No  1 Yes  88 Refused  99 Don’t know |  |  |
| **va_swe_abd_day** | How long before death did s/he have protruding abdomen (days) | 0-31 as reported  88 Refused  99 Don’t know | Less than 24 hours = 0 days |  |
| **va_swe_abd_month** | How long before death did s/he have protruding abdomen (months) | 1-60 as reported  88 Refused  99 Don’t know |  |  |
| **va_swe_abd_rapid** | How rapidly did s/he develop the protruding abdomen? | 0 Rapidly  1 Slowly  88 Refused  99 Don’t know |  |  |
| **va_mass_abd** | Any lump inside the abdomen | 0 No  1 Yes  88 Refused  99 Don’t know |  |  |
| **va_mass_abd_day** | How long did s/he have a mass in the abdomen (days) | 0-31 as reported  88 Refused  99 Don’t know | Less than 24 hours = 0 days |  |
| **va_mass_abd_month** | How long did s/he have a mass in the abdomen (months) | 1-60 as reported  88 Refused  99 Don’t know |  |  |
| **va_headache** | Any severe headache | 0 No  1 Yes  88 Refused  99 Don’t know |  |  |
| **va_skin** | Any skin problems | 0 No  1 Yes  88 Refused  99 Don’t know |  |  |
| **va_ulc_any** | Any ulcers or sores? | 0 No  1 Yes  88 Refused  99 Don’t know |  |  |
| **va_sores** | Any sores? | 0 No  1 Yes  88 Refused  99 Don’t know |  |  |
| **va_ulc_pus** | Did the sores have clear fluid or pus? | 0 No  1 Yes  88 Refused  99 Don’t know |  |  |
| **va_ulc_feet** | Any ulcers/ abscesses or sores on the feet | 0 No  1 Yes  88 Refused  99 Don’t know |  |  |
| **va_pus_feet** | Did the ulcer on the foot ooze pus? | 0 No  1 Yes  88 Refused  99 Don’t know |  |  |
| **va_pus_feet_day** | How long did the ulcer on the foot ooze pus (days) | 0-31 as reported  88 Refused  99 Don’t know | Less than 24 hours = 0 days |  |
| **va_pus_feet_month** | How long did the ulcer on the foot ooze pus (months) | 1-60 as reported  88 Refused  99 Don’t know |  |  |
| **va_ulc_oth** | Any ulcers/ abscesses or sores on body, apart from feet | 0 No  1 Yes  88 Refused  99 Don’t know |  |  |
| **va_ulc_breast** | Did she have any ulcers in the breast? | 0 No  1 Yes  88 Refused  99 Don’t know | Only for Women |  |
| **va_rash** | Any rash | 0 No  1 Yes  88 Refused  99 Don’t know |  |  |
| **va_rash_day** | For how many days did she have skin rash? | Number of days as reported  88 Refused  99 Don’t know | Less than 24 hours = 0 days |  |
| **va_rash_face** | Was there rash on the face? | 0 No  1 Yes  88 Refused  99 Don’t know |  |  |
| **va_rash_trunk** | Was there rash on the trunk/abdomen? | 0 No  1 Yes  88 Refused  99 Don’t know |  |  |
| **va_rash_extremities** | Was there rash on the extremities? | 0 No  1 Yes  88 Refused  99 Don’t know |  |  |
| **va_rash_all** | Was there rash everywhere? | 0 No  1 Yes  88 Refused  99 Don’t know |  |  |
| **va_measrash** | Any measles rash | 0 No  1 Yes  88 Refused  99 Don’t know |  |  |
| **va_herpes** | Any herpes zoster | 0 No  1 Yes  88 Refused  99 Don’t know |  |  |
| **va_skin_flake** | Did her/his skin flake off in patches? | 0 No  1 Yes  88 Refused  99 Don’t know |  |  |
| **va_stiff_neck** | Any stiff neck | 0 No  1 Yes  88 Refused  99 Don’t know |  |  |
| **va_stiff_neck_day** | How long before death did s/he have a stiff neck (days) | 0-31 as reported  88 Refused  99 Don’t know | Less than 24 hours = 0 days |  |
| **va_stiff_neck_month** | How long before death did s/he have a stiff neck (months) | 1-60 as reported  88 Refused  99 Don’t know |  |  |
| **va_pain_neck** | Did s/he have a painful neck during the illness that led to death? | 0 No  1 Yes  88 Refused  99 Don’t know |  |  |
| **va_pain_neck_day** | How long before death did s/he have a painful neck (days) | 0-31 as reported  88 Refused  99 Don’t know | Less than 24 hours = 0 days |  |
| **va_pain_neck_month** | How long before death did s/he have a painful neck (months) | 1-60 as reported  88 Refused  99 Don’t know |  |  |
| **va_any_coma** | Was s/he unconscious during the illness that led to death? | 0 No  1 Yes  88 Refused  99 Don’t know |  |  |
| **va_coma** | Was there a coma > 24hrs | 0 No  1 Yes  88 Refused  99 Don’t know |  |  |
| **va_coma_sudden** | Did the coma come on suddenly (at least within a single day) | 0 No  1 Yes  88 Refused  99 Don’t know |  |  |
| **va_coma_die** | Did the unconsciousness continue until death? | 0 No  1 Yes  88 Refused  99 Don’t know |  |  |
| **va_convul** | Any convulsions or fits | 0 No  1 Yes  88 Refused  99 Don’t know |  |  |
| **va_convul_min** | How many minutes did the convulsions last? | Minutes as reported  888 Refused  999 Don’t know | Less than 1 minute = 0 minutes |  |
| **va_convul_coma** | Became unconscious immediately after convulsions | 0 No  1 Yes  88 Refused  99 Don’t know |  |  |
| **va_urine** | Any abnormality of urine | 0 No  1 Yes  88 Refused  99 Don’t know |  |  |
| **va_uri_ret** | Any urinary retention | 0 No  1 Yes  88 Refused  99 Don’t know |  |  |
| **va_exc_urine** | Any excessive urination | 0 No  1 Yes  88 Refused  99 Don’t know |  |  |
| **va_uri_haem** | Any haematuria | 0 No  1 Yes  88 Refused  99 Don’t know |  |  |
| **va_wt_loss** | Any weight loss | 0 No  1 Yes  88 Refused  99 Don’t know |  |  |
| **va_wasting** | Any severe wasting | 0 No  1 Yes  88 Refused  99 Don’t know |  |  |
| **va_or_cand** | Any oral candidiasis | 0 No  1 Yes  88 Refused  99 Don’t know |  |  |
| **va_rigidity** | Any rigidity/lockjaw | 0 No  1 Yes  88 Refused  99 Don’t know |  |  |
| **va_lump** | Any localised lump or lesion | 0 No  1 Yes  88 Refused  99 Don’t know |  |  |
| **va_lump_mouth** | Any lump or lesion in mouth | 0 No  1 Yes  88 Refused  99 Don’t know |  |  |
| **va_lump_armpit** | Any lump or lesion in armpit | 0 No  1 Yes  88 Refused  99 Don’t know |  |  |
| **va_lump_neck** | Any lumps/swelling in neck | 0 No  1 Yes  88 Refused  99 Don’t know |  |  |
| **va_lump_breast** | Any breast lump or lesion | 0 No  1 Yes  88 Refused  99 Don’t know | Only for women |  |
| **va_lump_gen** | Any lump or lesion in groin or genitals | 0 No  1 Yes  88 Refused  99 Don’t know |  |  |
| **va_swe_face** | Any facial swelling/puffiness | 0 No  1 Yes  88 Refused  99 Don’t know |  |  |
| **va_swe_face_day** | How long did s/he have puffiness of the face (days) | 0-31 as reported  88 Refused  99 Don’t know | Less than 24 hours = 0 days |  |
| **va_swe_face_month** | How long did s/he have puffiness of the face (months) | 1-60 as reported  88 Refused  99 Don’t know |  |  |
| **va_swe_leg** | Any swollen legs or feet? | 0 No  1 Yes  88 Refused  99 Don’t know |  |  |
| **va_swe_leg_day** | How long did s/he have swelling of legs (days) | 0-31 as reported  88 Refused  99 Don’t know | Less than 24 hours = 0 days |  |
| **va_swe_leg_month** | How long did s/he have swelling of legs (months) | 1-60 as reported  88 Refused  99 Don’t know |  |  |
| **va_swe_ankles** | Any swelling of both feet/ankles | 0 No  1 Yes  88 Refused  99 Don’t know |  |  |
| **va_swe_all** | Did s/he have general puffiness all over his/her body? | 0 No  1 Yes  88 Refused  99 Don’t know |  |  |
| **va_anaemia** | Any anaemia/paleness | 0 No  1 Yes  88 Refused  99 Don’t know |  |  |
| **va_exc_drink** | Any excessive water intake | 0 No  1 Yes  88 Refused  99 Don’t know |  |  |
| **va_hair** | Any abnormal hair colouring | 0 No  1 Yes  88 Refused  99 Don’t know |  |  |
| **va_paralysis** | Was there paralysis | 0 no paralysis  1 one side  2 both sides  88 Refused  99 Don’t know |  |  |
| **va_para_right** | Was the right side paralysed? | 0 No  1 Yes  88 Refused  99 Don’t know |  |  |
| **va_para_left** | Was the left side paralysed? | 0 No  1 Yes  88 Refused  99 Don’t know |  |  |
| **va_para_lower** | Was the lower side of the body paralysed? | 0 No  1 Yes  88 Refused  99 Don’t know |  |  |
| **va_para_upper** | Was the upper side of the body paralysed? | 0 No  1 Yes  88 Refused  99 Don’t know |  |  |
| **va_para_leg** | Was one leg paralysed? | 0 No  1 Yes  88 Refused  99 Don’t know |  |  |
| **va_para_arm** | Was one arm paralysed? | 0 No  1 Yes  88 Refused  99 Don’t know |  |  |
| **va_para_body** | Was the whole body paralysed? | 0 No  1 Yes  88 Refused  99 Don’t know |  |  |
| **va_bleed_any** | Did s/he bleed from anywhere? | 0 No  1 Yes  88 Refused  99 Don’t know |  |  |
| **va_bleed** | Was the any bleeding from mouth, nose and anus | 0 No  1 Yes  88 Refused  99 Don’t know |  |  |
| **va_mens** | Did she ever have a period of menstruate? | 0 No  1 Yes  88 Refused  99 Don’t know | Women only |  |
| **va_menstrual** | Was there any bleeding between menstrual periods | 0 No  1 Yes  88 Refused  99 Don’t know | Women only |  |
| **va_mens_excess** | Was the bleeding between menstrual periods excessive? | 0 No  1 Yes  88 Refused  99 Don’t know | Women only |  |
| **va_bleed_excess** | Was there excessive vaginal bleeding in the week prior to death? | 0 No  1 Yes  88 Refused  99 Don’t know | Women only |  |
| **va_ menstr_stop** | Had the woman’s normal vaginal bleeding stopped naturally | 0 No  1 Yes  88 Refused  99 Don’t know | Women over 40 only |  |
| **va_menstr_due** | At the time of death was her period overdue? | 0 No  1 Yes  88 Refused  99 Don’t know | Women only |  |
| **va_menstr_week** | For how many weeks had her period been overdue? | Number of weeks as reported  88 Refused  99 Don’t know | Women only |  |
| **va_menstr_post** | Had the woman’s normal vaginal bleeding stopped naturally but they later experienced vaginal bleeding | 0 No  1 Yes  88 Refused  99 Don’t know | Women only |  |
| **va_sharp** | Did she have a sharp pain in her belly shortly before death? | Number of weeks as reported  88 Refused  99 Don’t know | Women only |  |
| **va_pain_swallow** | Did s/he have pain upon swallowing? | 0 No  1 Yes  88 Refused  99 Don’t know |  |  |
| **va_drink_diff_type** | Was the difficulty with swallowing with solids, liquids or both? | 0 Solids  1 Liquids  2 Both  88 Refused  99 Don’t know |  |  |
| **va_drink_diff** | Any difficulty or pain in swallowing liquids | 0 No  1 Yes  88 Refused  99 Don’t know | - " - |  |
| **va_drink_diff_day** | How long before death did s/he have difficulty swallowing (days) | 0-31 as reported  88 Refused  99 Don’t know | Less than 24 hours = 0 days |  |
| **va_drink_diff_month** | How long before death did s/he have difficulty swallowing (months) | 1-60 as reported  88 Refused  99 Don’t know |  |  |
| **va_preg_status** | Was she pregnant or did she deliver less than 6 weeks before she died | 0 reported not pregnant within last 6 weeks  1 pregnant at time of death  2 died < 6 weeks after normal length pregnancy  3 died < 6 weeks after early pregnancy ending (<6 months) | Stata code “.” if questions not asked or answers not known |  |
| **va_preg_1yr** | Was this a woman who died more than 6 weeks, but less than 1 year, after being pregnant or delivering a baby? | 0 No  1 Yes  88 Refused  99 Don’t know |  |  |
| **va_b_num** | How many births, including stillbirths, did the baby’s mother have before this baby? | Number as reported  88 Refused  99 Don’t know |  |  |
| **va_first_p** | Did she die during/just after first pregnancy | 0 No  1 Yes  88 Refused  99 Don’t know |  |  |
| **va_more4** | Did she have more than 4 previous pregnancies | 0 No  1 Yes  88 Refused  99 Don’t know |  |  |
| **va_cs_prev** | Any previous Caesarean section | 0 No  1 Yes  88 Refused  99 Don’t know |  |  |
| **va_multip** | Was this a multiple pregnancy | 0 No  1 Yes  88 Refused  99 Don’t know |  |  |
| **va_lb_hours** | How many hours did labour and delivery take? | Report in hours  88 Refused  99 Don’t know | If less than 1 hour then enter 0 |  |
| **va_lab_24** | Was labour prolonged > 24 hrs | 0 No  1 Yes  88 Refused  99 Don’t know |  |  |
| **va_died_lab** | Did she die in labour undelivered | 0 No  1 Yes  88 Refused  99 Don’t know |  |  |
| **va_died_postlab** | Did she die after delivering a baby? | 0 No  1 Yes  88 Refused  99 Don’t know |  |  |
| **va_death_24** | Death within 24 hrs of pregnancy ending | 0 No  1 Yes  88 Refused  99 Don’t know |  |  |
| **va_baby_al** | Did she deliver a live baby within 6 wks of death | 0 No  1 Yes  88 Refused  99 Don’t know |  |  |
| **va_breast_fd** | Was she breast feeding at death | 0 No  1 Yes  88 Refused  99 Don’t know |  |  |
| **va_delivery** | Where did delivery take place | 0 at home  1 in transit  2 at health facility  3 Hospital  4 Health centre  88 Refused  99 Don’t know |  |  |
| **va_prof_ass** | Had professional assistance at delivery | 0 No  1 Yes  88 Refused  99 Don’t know |  |  |
| **va_who_deliver** | Who delivered the baby? | 0 Doctor  1 Midwife  2 Nurse  3 Relative  4 Self (mother)  5 Traditional birth attendant  6 Other  88 Refused  99 Don’t know |  |  |
| **va_del_method** | How was the baby delivered? | 0 normal vaginal delivery, no instruments  1 vaginal delivery with forceps / Ventuse  2 delivery by Caesarean section |  |  |
| **va_baby_pos** | Was baby's delivery position abnormal | 0 No  1 Yes  88 Refused  99 Don’t know |  |  |
| **va_mon_early** | Was the baby born more than one month early? | 0 No  1 Yes  88 Refused  99 Don’t know |  |  |
| **va_preg_months** | For how many months was she pregnant? | 0 No  1 Yes  88 Refused  99 Don’t know |  |  |
| **va_hyster** | Hysterectomy shortly before death | 0 No  1 Yes  88 Refused  99 Don’t know | - " - |  |
| **va_bpr_preg** | Was blood pressure raised during pregnancy | 0 No  1 Yes  88 Refused  99 Don’t know |  |  |
| **va_fit_preg** | During the last 3 months of pregnancy, did she suffer from convulsions? | 0 No  1 Yes  88 Refused  99 Don’t know |  |  |
| **va_vis_bl_preg** | Any blurred vision during the last 3 months of preg | 0 No  1 Yes  88 Refused  99 Don’t know |  |  |
| **va_bleed_m** | Mother had excessive vaginal bleeding in pregnancy/postpartum period | 0 No  1 Yes  88 Refused  99 Don’t know |  |  |
| **va_bleed_preg** | Major bleeding in first 6 months of pregnancy | 0 No  1 Yes  88 Refused  99 Don’t know |  |  |
| **va_bleed_pre_lab** | Major bleeding during last 3 months of pregnancy but before labour | 0 No  1 Yes  88 Refused  99 Don’t know |  |  |
| **va_bleed_lab** | Major bleeding during labour, before delivering the baby | 0 No  1 Yes  88 Refused  99 Don’t know |  |  |
| **va_bleed_post_lab** | Major bleeding after delivering the baby or an abortion | 0 No  1 Yes  88 Refused  99 Don’t know |  |  |
| **va_placent_r** | Did placenta remain inside | 0 No  1 Yes  88 Refused  99 Don’t know |  |  |
| **va_disch_sm** | Any foul smelling vaginal discharge | 0 No  1 Yes  88 Refused  99 Don’t know |  |  |
| **va_term_att** | Any attempt to terminate this pregnancy | 0 No  1 Yes  88 Refused  99 Don’t know |  |  |
| **va_rec_abort** | Any recent abortion | 0 No  1 Yes  88 Refused  99 Don’t know |  |  |
| **va_abort** | Did she die during an abortion? | 0 No  1 Yes  88 Refused  99 Don’t know |  |  |
| **va_abort_post** | Did she die within 6 weeks after having an abortion? | 0 No  1 Yes  88 Refused  99 Don’t know |  |  |
| **va_injury** | Any obvious recent injury | 0 No  1 Yes  88 Refused  99 Don’t know |  |  |
| **va_transport _road** | Was s/he in a road transport accident | 0 No  1 Yes  88 Refused  99 Don’t know |  |  |
| **va_transport_role** | What was her/his role in the road traffic accident? | 0 Pedestrian  1 Driver or passenger in car or light vehicle  2 Driver or passenger in bus or heavy vehicle  3 Driver or passenger on motorcycle  4 Driver or passenger on pedal cycle  5 Other  88 Refused  99 Don’t know |  |  |
| **va_transport_counter** | What was the counterpart that was hit during the road traffic accident? | 0 Pedestrian  1 Stationary object  2 Car or light vehicle  3 Bus or heavy vehicle  4 Motorcycle  5 Pedal cycle  6 Other  88 Refused  99 Don’t know |  |  |
| **va_transport_oth** | Was s/he in a non-road transport accident | 0 No  1 Yes  88 Refused  99 Don’t know |  |  |
| **va_fall** | Had s/he fallen recently | 0 No  1 Yes  88 Refused  99 Don’t know |  |  |
| **va_drowning** | Did s/he drown | 0 No  1 Yes  88 Refused  99 Don’t know |  |  |
| **va_burn** | Was s/he burnt by heat, steam or fire | 0 No  1 Yes  88 Refused  99 Don’t know |  |  |
| **va_assault** | Injured in some kind of violence or assault by another person | 0 No  1 Yes  88 Refused  99 Don’t know | NB corrected spelling in variable name |  |
| **va_firearm** | Was s/he injured by a firearm? | 0 No  1 Yes  88 Refused  99 Don’t know |  |  |
| **va_stab** | Was s/he stabbed, cut or pierced? | 0 No  1 Yes  88 Refused  99 Don’t know |  |  |
| **va_strangle** | Was s/he strangled? | 0 No  1 Yes  88 Refused  99 Don’t know |  |  |
| **va_blunt** | Was s/he injured by a blunt force? | 0 No  1 Yes  88 Refused  99 Don’t know |  |  |
| **va_venom** | Any poisoning, bite, sting from a venomous animal or insect | 0 No  1 Yes  88 Refused  99 Don’t know |  |  |
| **va_nonvenom** | Any bite or sting from a non-venomous animal or insect | 0 No  1 Yes  88 Refused  99 Don’t know |  |  |
| **va_animal** | What was the animal/insect | 0 Dog  1 Snake  2 Insect or scorpion  3 Other  88 Refused  99 Don’t know |  |  |
| **va_nature** | Was s/he injured by a force of nature | 0 No  1 Yes  88 Refused  99 Don’t know |  |  |
| **va_electrocution** | Was it electrocution? | 0 No  1 Yes  88 Refused  99 Don’t know |  |  |
| **va_poison** | Was there any poisoning? | 0 No  1 Yes  88 Refused  99 Don’t know |  |  |
| **va_inj_other** | Did s/he encounter any other injury? | 0 No  1 Yes  88 Refused  99 Don’t know |  |  |
| **va_inj_accident** | Was the injury accidental? | 0 No  1 Yes  88 Refused  99 Don’t know |  |  |
| **va_inj_intent** | Was h/she intentionally injured by another person or people | 0 No  1 Yes  88 Refused  99 Don’t know |  |  |
| **va_suicide** | Any suggestion of suicide | 0 No  1 Yes  88 Refused  99 Don’t know |  |  |
| **va_alcohol** | Was s/he known to drink alcohol | 0 No  1 Yes  88 Refused  99 Don’t know |  |  |
| **va_tobacco** | Did s/he use tobacco? | 0 No  1 Yes  88 Refused  99 Don’t know |  |  |
| **va_smoking** | Was s/he a known smoker | 0 No  1 Yes  88 Refused  99 Don’t know |  |  |
| **va_cig** | Did s/he use cigarettes? | 0 No  1 Yes  88 Refused  99 Don’t know |  |  |
| **va_cig_num** | How many cigarettes did s/he smoke daily? | Number as reported  88 Refused  99 Don’t know |  |  |
| **va_pipe** | Did s/he use a pipe? | 0 No  1 Yes  88 Refused  99 Don’t know |  |  |
| **va_chew** | Did s/he use chewing tobacco? | 0 No  1 Yes  88 Refused  99 Don’t know |  |  |
| **va_loc** | Did s/he use a local form of tobacco? | 0 No  1 Yes  88 Refused  99 Don’t know |  |  |
| **va_tobac_num** | How many times did s/he use tobacco products per day | Number as reported  88 Refused  99 Don’t know |  |  |
| **va_married** | Was she married/partnered at death | 0 No  1 Yes  88 Refused  99 Don’t know |  |  |
| **va_vaccin** | Was s/he adequately vaccinated | 0 No  1 Yes  88 Refused  99 Don’t know |  |  |
| **va_treatment** | Treatment for final illness from a health facility | 0 No  1 Yes  88 Refused  99 Don’t know |  |  |
| **va_rehydrat** | Was oral rehydration required during final illness | 0 No  1 Yes  88 Refused  99 Don’t know |  |  |
| **va_iv** | Was an IV drip required during final illness | 0 No  1 Yes  88 Refused  99 Don’t know |  |  |
| **va_blood_tr** | Was blood transfusion required during final illness | 0 No  1 Yes  88 Refused  99 Don’t know |  |  |
| **va_nose** | Was treatment/food required through nose during final illness | 0 No  1 Yes  88 Refused  99 Don’t know |  |  |
| **va_antib_i** | Was antibiotic injection required during final illness | 0 No  1 Yes  88 Refused  99 Don’t know |  |  |
| **va_art** | Did s/he receive or need antiretroviral therapy? | 0 No  1 Yes  88 Refused  99 Don’t know |  |  |
| **va_surgery** | Any operation for the illness? | 0 No  1 Yes  88 Refused  99 Don’t know |  |  |
| **va_operation** | Was there an operation within one month of death | 0 No  1 Yes  88 Refused  99 Don’t know |  |  |
| **va_disch** | Was discharged from hospital very ill | 0 No  1 Yes  88 Refused  99 Don’t know |  |  |
| **va_out** | Was care sought outside the home while s/he has the illness? | 1 Yes  0 No  88 Refused  99 Don’t know |  |  |
| **va_out_trad** | Sought care from traditional healer | 0 No  1 Yes  88 Refused  99 Don’t know |  |  |
| **va_out_homeo** | Sought care from homeopath | 0 No  1 Yes  88 Refused  99 Don’t know |  |  |
| **va_out_relig** | Sought care from religious leader | 0 No  1 Yes  88 Refused  99 Don’t know |  |  |
| **va_out_hosp** | Sought care from government hospital | 0 No  1 Yes  88 Refused  99 Don’t know |  |  |
| **va_out_hc** | Sought care from government health centre | 0 No  1 Yes  88 Refused  99 Don’t know |  |  |
| **va_out_priv** | Sought care from private hospital | 0 No  1 Yes  88 Refused  99 Don’t know |  |  |
| **va_out_chw** | Sought care from community based practitioner associated with health system | 0 No  1 Yes  88 Refused  99 Don’t know |  |  |
| **va_out_tba** | Sought care from traditional birth attendant | 0 No  1 Yes  88 Refused  99 Don’t know |  |  |
| **va_out_privp** | Sought care from private physician | 0 No  1 Yes  88 Refused  99 Don’t know |  |  |
| **va_out_friend** | Sought care from friend/relative | 0 No  1 Yes  88 Refused  99 Don’t know |  |  |
| **va_out_pharm** | Sought care from pharmacy | 0 No  1 Yes  88 Refused  99 Don’t know |  |  |
| **va_shospf** | In the final days before death, did s/he travel to the hospital or health facility? | 0 No  1 Yes  88 Refused  99 Don’t know |  |  |
| **va_strans** | Did they use motorised transport to get to the hospital or health facility? | 0 No  1 Yes  88 Refused  99 Don’t know |  |  |
| **va_sadmit** | Were there any problems during admission to the hospital or health facility? | 0 No  1 Yes  88 Refused  99 Don’t know |  |  |
| **va_streat** | Were there any problems with the way they were treated (medical treatment, procedures, inter-personal attitudes, respect, dignity) in the hospital or health facility? | 0 No  1 Yes  88 Refused  99 Don’t know |  |  |
| **va_smedic** | Were there any problems getting medications, or diagnostic tests in the hospital or health facility? | 0 No  1 Yes  88 Refused  99 Don’t know |  |  |
| **va_smore2** | Does it take more than 2 hours to get to the nearest hospital or health facility from the deceased’s household? | 0 No  1 Yes  88 Refused  99 Don’t know |  |  |
| **va_sdoubt** | In the final days before death, were there any doubts about whether medical care was needed? | 0 No  1 Yes  88 Refused  99 Don’t know |  |  |
| **va_stradm** | In the final days before death, was traditional medicine used? | 0 No  1 Yes  88 Refused  99 Don’t know |  |  |
| **va_smobph** | In the final days before death, did anyone use a telephone or cell phone to call for help? | 0 No  1 Yes  88 Refused  99 Don’t know |  |  |
| **va_scosts** | Over the course of the illness, did the total costs of care and treatment prohibit other household payments? | 0 No  1 Yes  88 Refused  99 Don’t know |  |  |
| **Informant’s understanding of deceased HIV service use** | | |  |  |
| **va_went_to_htc** | Did the deceased ever receive testing and counselling for HIV? | 0 No  1 Yes  88 Refused  99 Don’t know |  |  |
| **va_last_test_result** | What was the last HIV test result of the deceased | 0 negative  1 positive  88 Refused  99 Don’t know |  |  |
| **va_last_hiv_test_when** | When did deceased have last HIV test? | 1 in last year  2 more than a year ago  3 Never  88 Refused  99 Don’t know |  |  |
| **va_last_hiv_test_year** | When did deceased have last HIV test? | Year 4 digits  8888 Refused  9999 Don’t know |  |  |
| **va_last_hiv_test_month** | When did deceased have last HIV test? | Month as integer 1 to 12  88 Refused  99 Don’t know |  |  |
| **va_referred_art_ever** |  | 0 No  1 Yes  88 Refused  99 Don’t know |  |  |
| **va_referred_art_when** |  | 1 in last year  2 more than a year ago  3 Never  88 Refused  99 Don’t know |  |  |
| **va_referred_art_year** |  | Year 4 digits  8888 Refused  9999 Don’t know |  |  |
| **va_referred_art_month** |  | Month as integer 1 to 12  88 Refused  99 Don’t know |  |  |
| **va_hivclinic_ever** | Whether deceased ever went to clinic/other service for HIV positive people | 0 No  1 Yes  88 Refused  99 Don’t know |  |  |
| **va_hivclinic_first_when** | When deceased first went to HIV clinic/service | 1 in last year  2 more than a year ago  3 Never  88 Refused  99 Don’t know |  |  |
| **va_ hivclinic_first _year** |  | Year 4 digits  8888 Refused  9999 Don’t know |  |  |
| **va_ hivclinic_first_month** |  | Month as integer 1 to 12  88 Refused  99 Don’t know |  |  |
| **va_hivclinic_current** | Was deceased attending HIV clinic/service around the time of death, were they still a patient. | 0 No  1 Yes  88 Refused  99 Don’t know |  |  |
| **va_assessed_for_art** | Was deceased ever assessed for ART treatment need? | 0 No  1 Yes  88 Refused  99 Don’t know |  |  |
| **va_first_assessed_when** |  | 1 in last year  2 more than a year ago  3 Never  88 Refused  99 Don’t know |  |  |
| **va_first_assessed_year** |  | Year 4 digits  8888 Refused  9999 Don’t know |  |  |
| **va_first_assessed_month** |  | Month as integer 1 to 12  88 Refused  99 Don’t know |  |  |
| **va_ever_prescribed_art** | Was the deceased ever prescribed ART treatment? | 0 No  1 Yes  88 Refused  99 Don’t know |  |  |
| **va_started_art** | Did deceased start ART treatment? | 0 No  1 Yes  88 Refused  99 Don’t know |  |  |
| **va_date_start_art** | When did the deceased start on ART treatment? | Date in Stata format |  |  |
| **va_art_current** | Was the deceased still on ART treatment when they died? | 0 No  1 Yes  88 Refused  99 Don’t know |  |  |
| **va_date_stop_art** | When did the deceased stop ART treatment? | Date in Stata format |  |  |
| **va_where_art** | Where did the deceased receive ART treatment? | 0 not treated  1 local clinic  2 clinic outside study area  3 not known  88 Refused  99 Don’t know |  |  |
